# The psychophysics of dynamic gaze-following saccades during search

**DOI:** 10.1167/jov.25.14.14

**Published:** 2025-12-17

**Authors:** Srijita Karmakar, Miguel P. Eckstein

**Affiliations:** 1 Department of Psychological and Brain Sciences, University of California, Santa Barbara, USA

**Keywords:** gaze cueing, overt attention, saccades

## Abstract

The ability to quickly and precisely follow another person's gaze reflects critical evolutionary mechanisms underlying social interactions, such as attention modulation and the prediction of others’ future actions. Recent studies show that observers use another person's gaze direction and peripheral scene information to make anticipatory saccades toward the gaze goal. However, it remains unclear how these eye movements are influenced by complex features of natural scenes, such as a foveal gazer, multiple peripheral gaze goals, and the relative distance between gazer and goal. We presented dynamic stimuli (videos) of real-world scenes with or without a gazer shifting their head to gaze at other individuals (gaze goals). Participants were instructed to search for a specific target individual in the videos while their eye movements were recorded. We measured the accuracy of the first saccade in locating the gaze goal. First, we found that the absence of a foveal gazer significantly increased saccade error, but only when the goal was at least approximately 9 degrees of visual angle from the initial fixation. First saccade amplitude and onset latency were higher in the gazer-present condition. Second, when there were multiple potential gaze goals in the periphery, the first saccade was directed to the individual closer to the initial fixation (gazer) location. Finally, the presence of multiple peripheral gaze goals shortened saccade latencies and increased the frequency of anticipatory saccades made before the gazer completed their head movement. These findings extend our understanding of gaze following in complex, naturalistic scenes and inform theories of attention and real-world decision-making.

## Introduction

Visual search is an important perceptual process that is recruited in almost every activity required for an organism's survival, such as foraging for food, looking for a friend among a crowd of people, or searching for apartment keys on a cluttered table. For humans and most other nonhuman primates, foveated vision plays a major role in guiding visual search (Bringmann et al., 2018; Chen, Hoffmann, Distler, & Ziad, 2019; Mustari, 2017). The human eye does not process all parts of an image equally: the fovea is a small central area of our vision with the greatest visual acuity, while the visual periphery suffers from increasing degradation in spatial resolution. Owing to the varying spatial detail in information processing across the visual field, humans rely heavily on eye movements to continually shift the fovea to important regions in the visual scene (see Kowler, 2011; Spering, 2022 for reviews). To this end, humans make approximately three ballistic eye movements, or saccades, in just 1 second (Andrews & Coppola, 1999; Gibaldi & Sabatini, 2021; Land, Mennie, & Rusted, 1999). Given their constant necessity in daily tasks, eye movements thus make for an important and easy-to-access measure of human visual and perceptual behavior (Eckstein, 2017; Huang, Andrist, Sauppé, & Mutlu, 2015; Park, Lee, Lee, Chang, & Kwak, 2016; Ibbotson & Krekelberg, 2011). The brain undertakes fine-grained computations that incorporate stimulus properties, combined with the foveated nature of the visual system, to guide eye movements toward task-relevant visual information in tasks such as reading (Legge, Klitz, & Tjan, 1997; Strandberg, Nilsson, Ostberg, & Seimyr, 2023), visual search (Droll, Abbey, & Eckstein, 2009; Eckstein, Beutter, & Stone, 2001; Eckstein, Schoonveld, Zhang, Mack, & Akbas, 2015; Findlay, 1995; Findlay, 1997; Han & Eckstein, 2023a; Najemnik & Geisler, 2005; Neider & Zelinsky, 2006), face perception and recognition (Chuk, Chan, & Hsiao, 2014; Han, Chakravarthula, & Eckstein, 2021; Peterson & Eckstein, 2012), object recognition (Leek at al., 2012; Poth, Herwig, & Schneider, 2015; Rucci, Iovin, Poletti, & Santini,2007), scene understanding (Castelhano & Heaven, 2011; Torralba, Oliva, Castelhano, & Henderson, 2006), motion perception (Beutter & Stone, 1998; Spering, Schütz, Braun, & Gegenfurtner, 2011), and motor actions (Hayhoe, Shrivastava, Mruczek, & Pelz, 2003; Hayhoe & Ballard, 2005; also see Schütz, Braun, & Gegenfurtner, 2011; Spering, 2022, for reviews).

In the real world, the human visual system is constantly bombarded with visual information, usually comprising task-relevant and task-irrelevant items. For instance, for a person driving a car, traffic signals and other cars on the road are relevant to the task of driving safely. However, the same scene can contain task-irrelevant details such as a large and colorful billboard or a bird sitting atop the traffic signal post. In such scenarios, the visual system has to select the task-relevant regions to attend to by utilizing various cues to guide attentional processing. For real-world scenes, overt attention toward visual search targets is guided by scene context such as a background and objects co-occurring with the target (Castelhano & Heaven, 2011; Koehler & Eckstein, 2017a; Neider & Zelinsky, 2006; Torralba et al., 2006; Võ, 2021), which are used as visual cues. In controlled experimental settings, previous studies have demonstrated that observers viewing simplified laboratory stimuli also use predictive cues of a target location to orient and guide covert attention and overt eye movements (Baek, Dosher, & Lu, 2021; Carrasco & Yeshurun, 1998; Eckstein, 2017; Eckstein, Pham, & Shimozaki, 2004; Liu, Xu, Yang, Li, & Huang, 2022; Lu & Dosher, 2000; Luck et al., 1994; Posner, 1980; Tünnermann & Scharlau, 2016; Zimmer et al., 2022).

The gaze of another person is one such important and socially informative real-world cue used by observers to orient attention and facilitate search (Baron-Cohen, 1995; Driver et al., 1999; Friesen, Ristic, & Kingstone, 2004; Han & Eckstein, 2022; Han & Eckstein, 2023a; Mareschal, Calder, & Clifford, 2013; Mareschal, Calder, Dadds, & Clifford, 2013). The perception of gaze has been extensively studied using psychophysics to reveal some of its key features. For instance, Gamer and Hecht (2007) measured the range (cone) of gaze directions perceived as looking at oneself, and Horstmann and Linke (2025) recently showed that the perception of two-dimensional gaze is distorted at smaller distances, whereas three-dimensional gaze perception has a small but reliable overestimation. Gaze perception also results in reliable adaptation aftereffects, akin to perceiving basic visual features like color and motion (see Clifford & Palmer, 2018 for a review).

The ability to follow another person's gaze direction quickly and precisely reflects important mechanisms that underlie social interactions, such as attention modulation and prediction of future actions (Baron-Cohen, 1995; Driver et al., 1999; Frischen, Bayliss, & Tipper, 2007). Perception of another person's gaze direction is influenced not only by the position of the iris in the gazer's eye but also by the gazer's head orientation (Nummenmaa & Calder, 2009; Todorović, 2006). Eye gaze and head direction, although similar in that both orient the observer's attention, differ in important ways: eye gaze is more difficult to perceive further out in the periphery than head direction (Loomis, Kelly, Pusch, Bailenson, & Beall, 2008). Thus, head direction is more reliable as a gaze cue even at very large eccentricities. Our study used two-dimensional stimuli (videos) and the viewing angles subtended by the people in the stimulus videos corresponded to viewing distances where eye gaze provided little information and head direction was a more reliable gaze cue. Despite the perceptual differences between the two, such as head direction not showing a positive serial dependency effect, unlike eye direction (Alais, Kong, Palmer, & Clifford, 2018), both head and eye gaze work independently to mutually influence and direct social attention (Langton, 2000; Nummenmaa & Calder, 2009; Todorović, 2006), also called joint attention and gaze following (Rayson, Bonaiuto, Ferrari, Chakrabarti, & Murray, 2019; Stephenson, Edwards, & Bayliss, 2021; see Shepherd, 2010 for a review).

Although gaze following is an automatic and reflexive phenomenon (Brooks & Meltzoff, 2005; Shepherd, 2010; Wallis et al., 2015), and have been previously modelled as a Bayesian inference process (Mareschal et al., 2013), the mechanisms behind how the brain achieves this quick and accurate inferential response are still not understood (see Itier & Batty, 2009; Nummenmaa & Calder, 2009 for reviews). Previous studies of gaze perception and gaze following have found that gaze cues orient reflexive covert and/or overt attention (Driver et al., 1999) and covert attention (Friesen et al., 2004) as measured by a reduction in reaction time when the target appears on the cued side. Driver et al. (1999) also showed that gaze cues, although presented centrally, reflexively orient the observer's attention like transient peripheral cues. Foveally presented gaze cues elicit behavioral signatures of automaticity and inhibition of return at longer stimulus onset asynchrony, similar to peripherally presented synthetic cues, such as boxes and flashes (Frischen et al., 2007). In contrast with exogenous cueing, however, gaze cues have a slower rise in facilitation effects as well as a delayed onset of inhibition of return (Frischen et al., 2007). Murray, Drake, and Klein (2023) reported gaze-cueing effects that were most like a hybrid of exogenous and endogenous attentional orientation. Several studies in humans and macaques (Friesen et al., 2004; Ramezanpour & Fallah, 2022) also suggest an initial reflexive orientation by gaze cues, only later followed by a more top-down, volitional guidance.

These previous studies of gaze cueing have typically used static images or cartoon drawings of faces and/or eyes as an operationalization of gaze. However, the real world is dynamic, and especially so for the perception of gaze. For instance, Farroni, Johnson, Brockbank, and Simion (2000) showed that infants do not follow eye gaze if presented statically, but do so only when there is apparent motion. In the same vein, Nummenmaa and Calder (2009) rightly highlight the importance of using dynamic and naturalistic stimuli to study social attention. To this end, a recent study from our lab used dynamic and real-world stimuli and found that observers use a gazer's head direction presented in the observer's fovea (henceforth referred to as foveal gazer) and peripheral information about potential gazed locations or objects (henceforth referred to as gaze goals) to execute anticipatory saccadic eye movements (Han & Eckstein, 2023b). Specifically, even before the video's gazer finishes orienting their head, the observers start eye movements toward the gazed location. This recent study had investigated relatively simple scenes with a foveal gazer always present, and with either a single peripheral gaze goal or none.

However, real-world scenes can contain multiple potential gaze goals that could appear at various distances from the gazer. For example, imagine you are talking to your friend (A) at a dinner party, and you are both looking for another friend (B). Suddenly, friend A turns their head to look toward a group of people. In this case, who your friend is looking at (their gaze goal) may be ambiguous. The gazer (friend A) may be looking at the searched target (friend B) or at another distractor person. How does our visual system make decisions about the gaze goal in such a scenario? In addition, the previous study evaluated the contribution of the peripheral gaze goal information in guiding observers’ saccades, but did not manipulate the presence of the gazer to delineate the influence of foveal information on observers’ saccade planning.

With this goal in mind, we aimed to use dynamic, naturalistic stimuli, and measure eye movements as a window to the inferential mechanisms used by the brain to perform gaze following during visual search for a target person. In the current study, we build on prior work by examining how humans follow gaze in naturalistic, dynamic scenes that include multiple potential gaze goals (either the target or a distractor person), a context that better reflects real-world social interactions. Crucially, we investigate how foveal and peripheral information contribute to observers’ anticipatory eye movements during gaze following. By manipulating the presence of a foveal gazer, the number of potential peripheral gaze goals, and the spatial distance between the gazer and the potential gaze goals, we aim to dissect the visual and cognitive mechanisms supporting rapid gaze-based inference. Participants viewed videos of real-world scenes with or without people (gazer) displaying gazing behavior at other individuals (likely gaze goals) present in the scene. Participants were not instructed to follow gaze cues, but simply to search for a previously specified target person, allowing us to observe spontaneous gaze-following behavior. To be clear, this target person could be either the goal or the non-goal in each trial. In other words, the gazer's head direction is uninformative about the target's location.

We hypothesize that 1) the presence of a foveal gazer will lead to slower saccade onsets owing to increased processing time and this processed gaze information will result in more accurate saccadic estimates of the goal location (Han & Eckstein, 2023b); 2) multiple potential gaze goals will increase spatial uncertainty about true gaze goal location compared to videos with only one goal present, and this will increase the first saccade end point error in estimating the true goal location; and 3) observers will initiate saccades toward potential gaze goals before the gazer's head movement ends (Han & Eckstein, 2023b), reflecting a predictive, inference-based mechanism that relies on both foveal and peripheral information available. We further predict that this anticipatory behavior will scale with the degree of peripheral uncertainty introduced at trial onset.

Together, these investigations address a fundamental question in visual cognition: how does the brain integrate foveal and peripheral information to make rapid, socially relevant inferences in visually complex environments? By systematically manipulating the presence of foveal gazer and peripheral gaze goal information, we evaluate the contributions of different visual inputs (foveal and peripheral) to anticipatory saccade planning. This design allows us to measure gaze-following behavior and test how visual uncertainty modulates predictive eye movements in a naturalistic search task.

## Methods

### Participants

The materials and procedures used in this study were approved by the University of California Internal Review Board. Thirty undergraduate students (9 males, 21 females; aged 18–23 years; mean, 19.67 *±* 1.27 years) from the University of California were recruited to participate in the experiment for research credits. All participants signed a consent form to provide informed consent to participate in the study. Participants were right handed and had normal or corrected-to-normal vision.

### Materials

**Figure 1.**
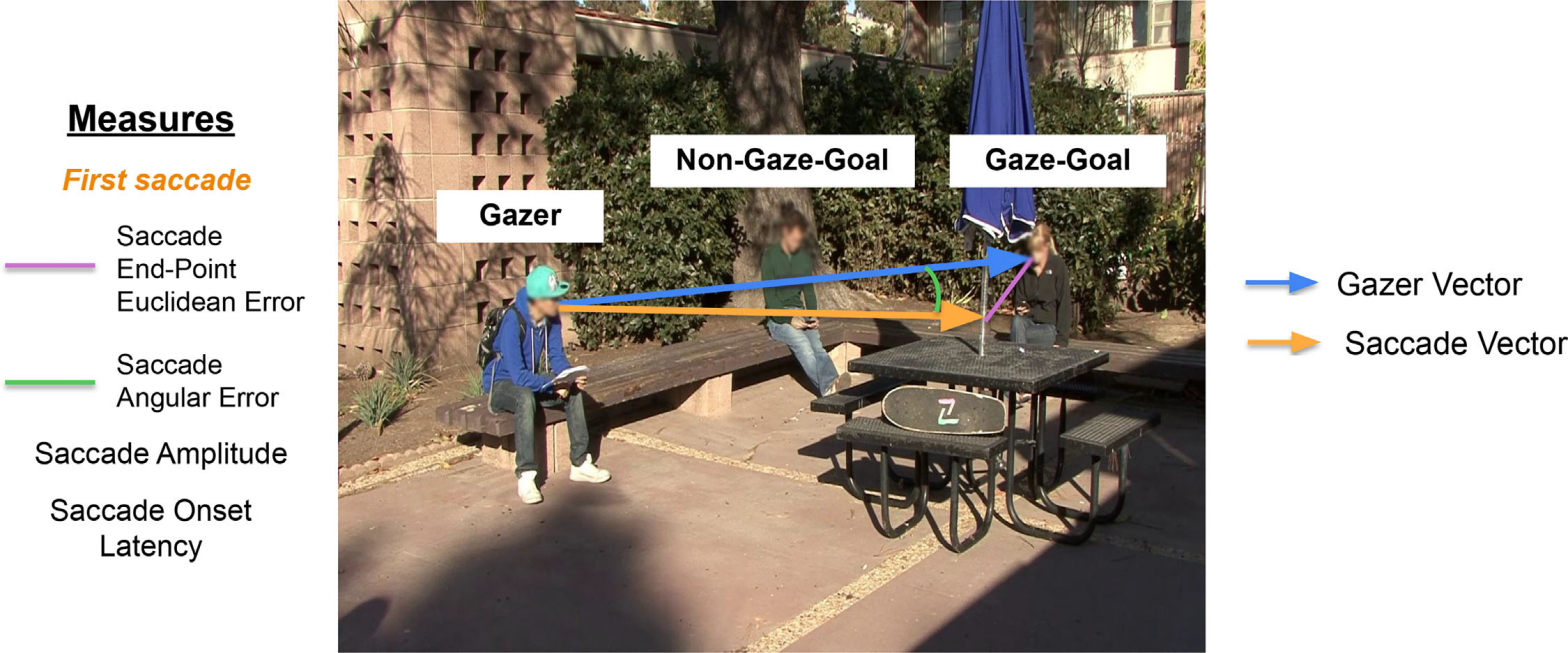
Terms used to define the experimental role of people in the videos and the DVs analyzed in this study. In this image, the gaze goal is a distractor individual, and the non-gaze goal is the target person (shown in Figure 4). The blue arrow shows the gazer vector or the gaze direction, defined as the line joining the gazer and the gaze direction end point. The orange arrow shows the saccade vector, defined as the line joining the start and end point of a given saccade. The DVs analyzed are as follows. 1) First saccade end point Euclidean error (shown as the purple line); this is the saccade error in estimating the gaze direction of the gazer. 2) First saccade angular error (shown as the green line). 3) First saccade amplitude (length of the orange arrow). 4) First saccade latency (time delay between stimulus onset and first saccade onset). Faces in the figure are blurred to protect the privacy of individuals; however, faces were displayed unaltered to the participants of the study.

Twenty-four unique in-house videos were used to create the stimuli for this study. The videos were recorded at the University of California, Santa Barbara in different indoor and outdoor settings (classrooms, cafeterias, outdoor campus, etc.). Each video contained multiple people. During filming, verbal instructions were given to one of the actors in the scene (the gazer) to look at another person (the gaze goal). At the start of the recording, only the background (without any actors) was recorded for a few seconds to aid with the later editing process. Next, once all the actors had entered the scene, the gazer individual was instructed to first look down and then, on a verbal cue from the scene director, start moving their head direction toward the goal individual. For the experiment, the videos were edited to begin when the gazer individual starts moving their head and end once the gazer finally stops their head movement to look at the gazed person (goal) who could either be the target or a distractor. The videos used for the gazer absent condition were the same ones except they were digitally edited to remove the gazer individuals and replace them with the scene background pixels. Thus, both gazer-absent and gazer-present counterparts of a given video had the same duration. There was one target person throughout the study: the person the observers were instructed to search for during the experimental task. All other individuals in the videos besides the target person and the gazer individual are referred to as distractors. Further, the individual that the gazer was instructed to look at during the filming of the videos is referred to as the gaze goal, and all other individuals whom the gazer does not look at are referred to as the non-gaze goals (Figure 1; note that, for privacy protection, faces are blurred in all figures here but were unaltered during the experiment). Importantly, the target person could be present as either the gaze goal or the non-gaze goal in a given trial. The videos were filmed on different days and in different settings, and as such, the target and distractor individuals appeared in different clothing and at varying distances from the gazer in each video. The gazer person could change across video clips. The average distance between the gazer and gaze goal across all trials in the study was 9.07 *±* 3.85 degrees of visual angle (dva).

To create the stimuli from the above videos, first, the 24 videos were reflected along the vertical axis to generate a total of 48 videos. We planned to include three experimental conditions in the study: gaze goal absent, one gaze goal, and two potential gaze goals. The original videos each had three individuals (one gazer, one gaze goal, and one non-gaze goal). To create the gaze goal absent condition, the gaze goal and non-gaze goal individuals were digitally erased using Adobe Premiere Pro by replacing the RGB values of the individuals with those of the background. Specifically, we used Adobe Premier Pro to manually select the individual(s) to be removed on each video and then replace the pixels within the selected regions with the background pixels grabbed from the early frames of video, when the actors had not entered the scene yet. Thus, the gaze goal absent (or no gaze goals) condition only includes the foveal gazer and no peripheral individuals. The one gaze goal condition was created by digitally removing the non-gaze goal from each video. For the two potential gaze goals condition, 6 of the 48 videos had the target individual as the gaze goal, and the non-gaze goal was a distractor. We created six more videos from the initial six videos by digitally swapping the target and distractor locations. This was done to allow us to compare eye movement features between trials in which the target individual appeared as the gaze goal and those where the target was the non-gaze goal, while all other features of the stimuli (such as gazer location, background, etc.) remained the same. Thus, the gazer present condition had 48 gaze goal absent videos, 48 one gaze goal videos wherein the target individual was the gaze goal in 12 of the videos and the remaining 36 videos had a distractor individual as the gaze goal, and 48 two potential gaze goals videos wherein 6 videos had the target individual as the gaze goal (and a distractor as a non-gaze goal), 6 others had the target individual as the non-gaze goal (and a distractor as the gaze-goal), and 36 videos had two distractors, one as the gaze goal and the other as non-gaze goal. This was done to maintain a 25% prevalence rate of the target individual in both the one gaze goal and the two potential gaze goal conditions.

**Figure 2.**
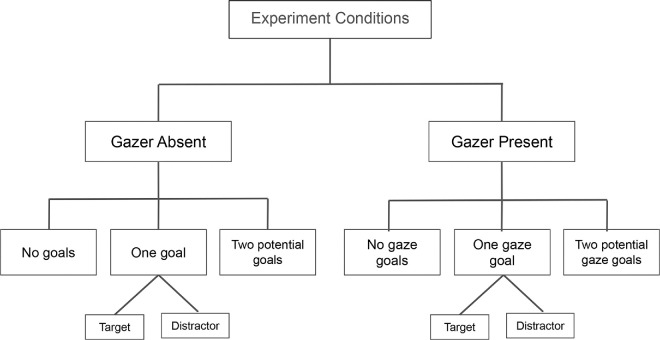
Experiment conditions. The flowchart shows the various sub conditions of the main experimental manipulations. Further subdivisions of the two potential gaze goals condition are described in Figure 3.

**Figure A1.**
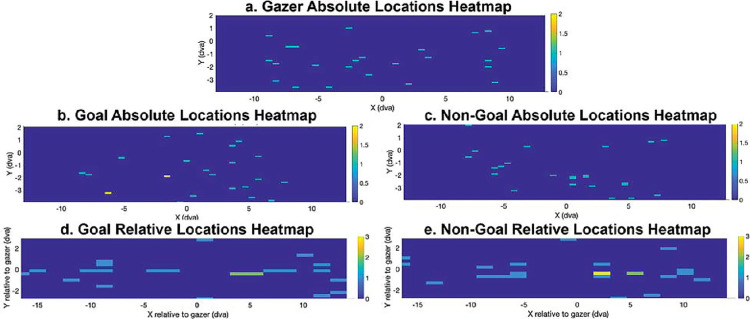
Location distribution heatmap of absolute and relative locations of stimulus individuals (gazer, goal, and non-goal) across our 24 unique videos. (**a**) Absolute location of gazer in dva with respect to screen center (0,0). (**b**) Absolute location of goal (dva to screen center). (**c**) Absolute location of non-goal (dva to screen center). (**d**) Relative location of goal with respect to gazer (dva). (**e**) Relative location of non-goal with respect to gazer (dva). Across trials, there is more variability in the location of the gazer, goal, and non-goal along the horizontal axis than along the vertical axis. Importantly, the heatmap of the absolute distributions show the absence of a center bias in gazer, goal, and non-goal locations.

Further, the gazer absent condition videos were created by digitally erasing the gazer individuals from each video used for the gazer present condition. Thus, the gazer absent condition had all the same videos as the gazer present condition (Figure 2). The gaze goal and non-gaze goal individuals are referred to as the goal and non-goal individuals for the gazer absent counterparts henceforth. Hence, in total, there were 48 (original videos) *×* 3 (goal condition: none, one goal, and two potential goals) *×* 2 (gazer condition: absent and present) = 288 total stimulus videos (trials). See Appendix Figures A1 through A4 for more details about our stimulus set.

All videos were presented at the center of an Acer monitor with 1,920 *×* 1,080 resolution subtending a visual angle of 31.5° *×* 19.0° (width *×* height) at a refresh rate of 60 Hz. The observers were seated at 980 mm from the monitor with their chins resting on a headrest while they watched the videos (0.017 dva per pixel). Stimulus presentation and data acquisition were controlled via Psychtoolbox 3.0.18 (Brainard, 1997; Kleiner et al., 2007; Pelli, 1997). Each observer's right eye was tracked using a video-based eye tracker, EyeLink 1000 Plus desktop mount (SR Research, Ontario, Canada) at a sampling rate of 1,000 Hz. Calibration and validation procedures were carried out using a 9 *×* 9 grid mandatorily at the start of every block as well as at the start of a trial if the recorded eye position was more than 1.5 dva away from the actual fixation location. Eye movement events with a velocity of greater than 30°/s and acceleration of greater than 8,000°/s^2^ were recorded as saccades.

### Design

**Figure 3.**
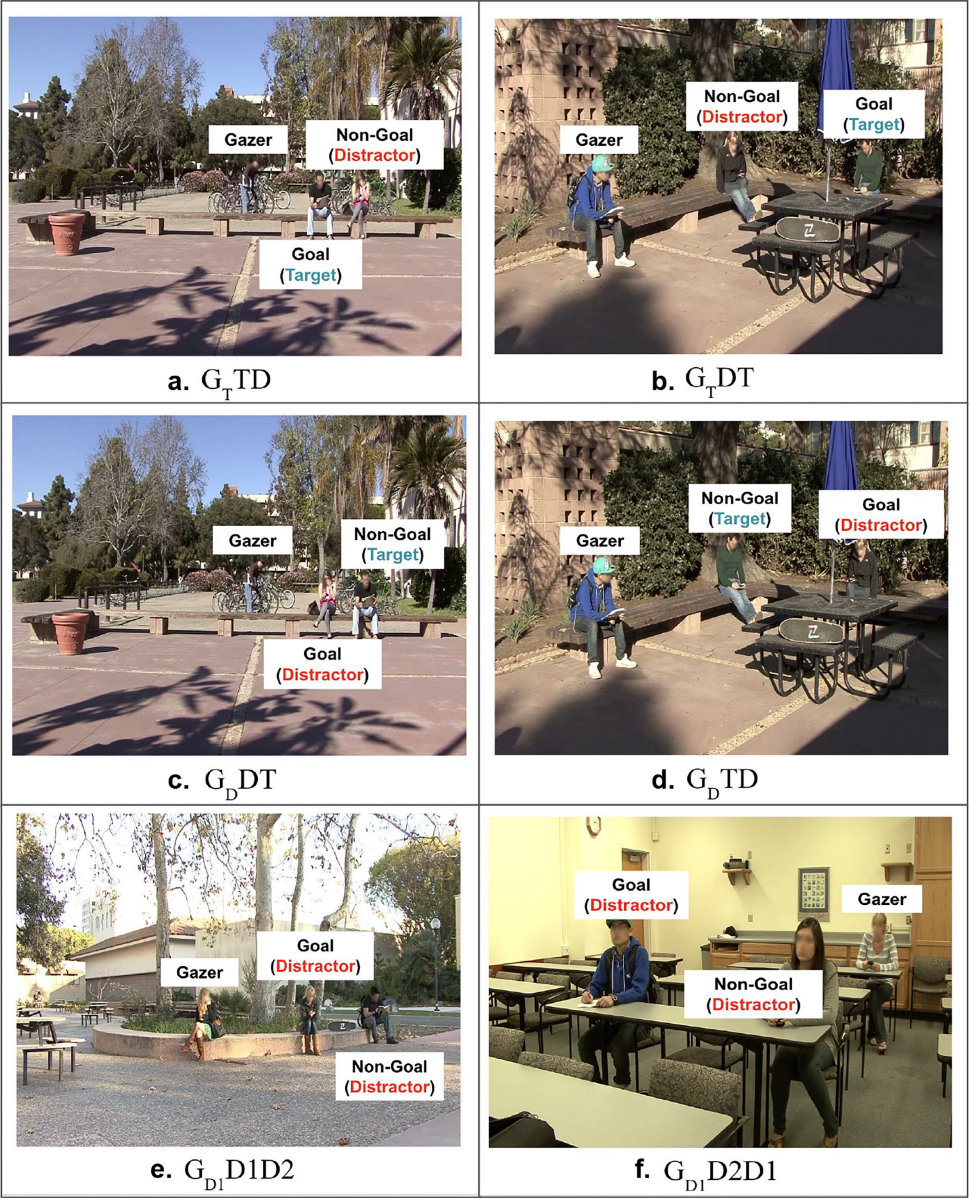
Sub-conditions of the two potential gaze goals condition (D, distractor; G, gazer; T, target). The subscripts in the condition label indicate the category of the goal, and the order of letters are in order of closeness to the initial fixation/gazer location. (**a**) Goal target and closer; non-goal distractor and farther (G_T_TD). (**b**) Goal target and farther; non-goal distractor and closer (G_T_DT). (**c**) Goal distractor and closer; non-goal target and farther (G_D_DT). (**d**) Goal distractor and farther, non-goal target and closer (G_D_TD). (**e**) Goal and non-goal distractors; goal closer (G_D1_D1D2). (**f**) Goal and non-goal distractors; goal farther (G_D2_D1D2). Faces in the figure are blurred to protect the privacy of individuals; however, faces were displayed unaltered to the participants of the study.

The study had a within-subjects design with two main conditions: gazer absent and gazer present, each of which had the same three sub-conditions: no gaze goal, one gaze goal, and two potential gaze goals (Figure 2). To be clear, we refer to the conditions in terms of gaze goals, although for the gazer absent condition, the goal individuals are present without the gazer. The target individual was present, either as the gaze goal or the non-gaze goal in 25% of all gaze goal present trials. The two potential gaze goals condition had three sub-conditions (see Figure 3): A) target as gaze goal, distractor as non-gaze goal, B) distractor as gaze goal, target as non-gaze goal, and C) distractors as both gaze goal and non-gaze goal.

The independent variables in the study are the following: A) presence of gazer, B) distance between initial fixation and gaze goal location, C) identity of gaze goal (target vs distractor), and D) number of potential gaze goals (no vs one vs two gaze goals). In addition, there were two further sub-conditions for the two potential gaze goals condition: A) gaze goal closer to initial fixation (also gazer location in gazer present trials) (e.g., Figures 3a, 3c, and 3e) and B) non-gaze goal closer to initial fixation (which is also the gazer location in gazer present trials) (e.g., Figures 3b, 3d, and 3f).

The dependent variables (DVs) in the study were the following: A) First saccade end point error, defined as the Euclidean distance between the actual gaze goal location and the landing point of the first saccade, B) First saccade angular error, defined as the angular displacement between the first saccade vector and the initial fixation (or gazer in gazer present trials) to gaze goal vector, C) First saccade onset latency, and D) First saccade amplitude (Figure 1). Specifically for gazer present trials, we also analyzed the gazer-head angular error at the time of initiation of the first saccade as a function of the number of potential gaze goals in the trial, as a measure of the observer's anticipation of the gazer's final head direction. We note the measures of error, both Euclidean and angular, relate to the two-dimensional videos presented to the observers and not the original three-dimensional scenes that were captured and projected onto the two-dimensional videos.

### Procedure

The study was carried out over 2 different days with an intervening period of 1 week between the 2 days. Observers were presented only with either gazer absent or gazer present trials on a given day. The order in which observers saw gazer absent and gazer present blocks was randomized across observers, such that 15 observers completed the gazer absent trials on the first day (and the gazer present trials on the second day), whereas the other 15 observers completed the gazer present trials on the first day. On visual inspection of the data, there were no differences in any of the DVs as a function of the order in which the conditions were presented, thus, we report our findings after combining data across all 30 observers.

**Figure 4.**
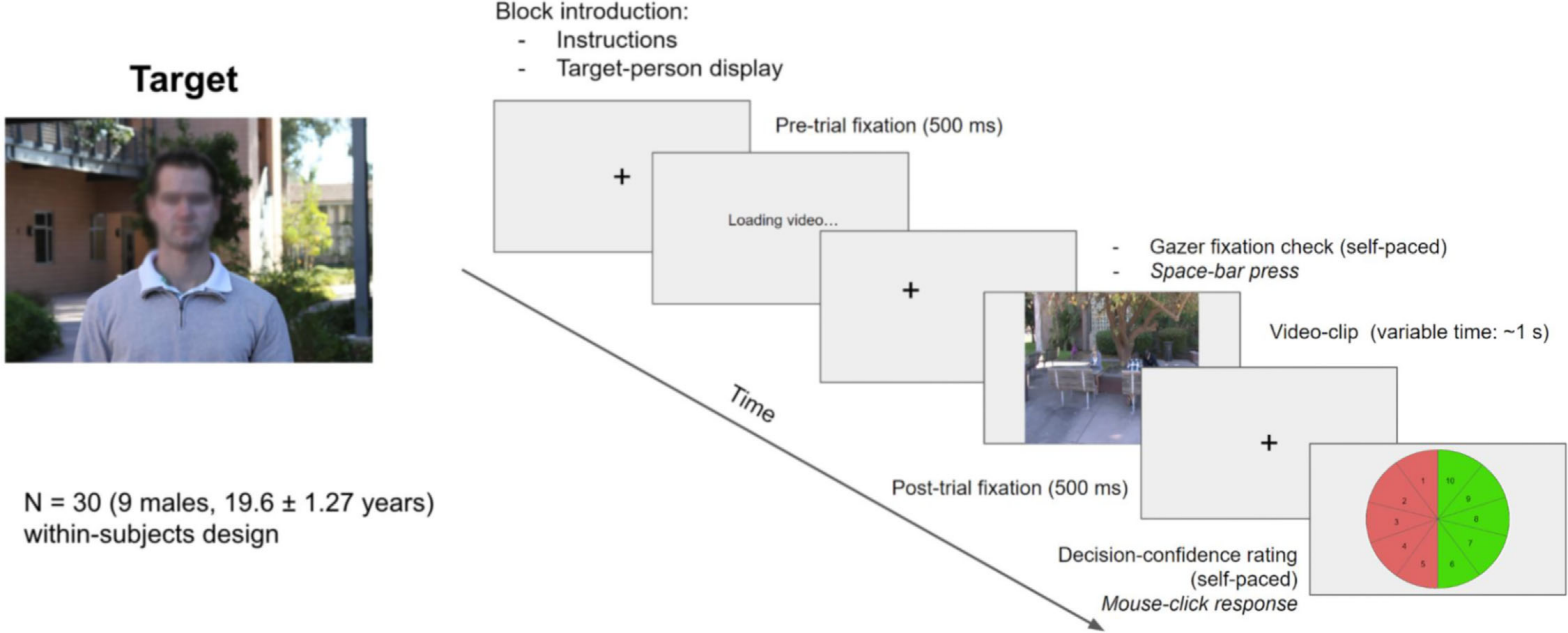
Experiment paradigm. Each trial started with a forced fixation that would display the gazer's head in gazer present trials after 500 ms, after which the trial started for variable duration (1.18 ± 0.32 seconds), at the end of which the observers were presented with a self-paced response screen of a wheel marked 1 through 10 (1–5, target absent; 6–10, target present; 1 and 10 being highly confident choices and 5 and 6 being highly uncertain choices), after which the screen moved on to the next trial. Each participant completed 144 trials (6 blocks of 24 trials) on 2 different days a week apart, thus completing 288 trials in total. Faces in the figure are blurred to protect the privacy of individuals; however, faces were displayed unaltered to the participants of the study.

On the first day, the participants gave informed consent. Observers were instructed to search for a specific target person in all the trials. They were not explicitly instructed to follow the gazer's direction of gaze. Participants completed 6 blocks of 24 trials (144 trials in total) each on a given day. At the start of each block, the participants conducted the eye tracker calibration and validation procedure. Two images of the target person they would be searching for were displayed at the start of every block, including the practice block. Before the very first block, participants completed a practice block of four trials comprising videos separate from the ones shown in the main experimental blocks. Next, the main experiment started with the first block of 24 trials. At the start of each trial, a fixation cross appeared on the screen over a gray image at the same location where the gazer's head would appear in a gazer present trial. Participants were required to fixate on the cross and if the recorded eye position was more than 1.5 dva away from the center of the fixation cross, participants were required to recalibrate and revalidate the eye tracker. Once the fixation check was passed, participants were shown the video trial which started at the frame in which the gazer's head movement started. The video ended at the frame in which the gazer's head movement stopped at the final gaze direction. On average, the duration of the stimuli videos was 1.18 *±* 0.32 seconds. After a trial ended, participants were presented with a response screen with a response wheel of numbers 1 through 10. They were instructed to click on a number between 1 and 5 if they thought the target was absent (1 being highly certain and 5 being highly uncertain) or to click on a number between 6 and 10 if they thought the target person was present (6 being highly uncertain and 10 being highly certain). Once the participants responded, the display moved on to the next trial which again started with a fixation cross. Participants completed 24 trials in each of the 6 blocks and returned on the next day to complete another 6 blocks of 24 trials each. The procedure remained the same on the second day except the participants did not complete any further practice trials (Figure 4).

### Human-estimated gaze information

One analysis of interest is to understand the inherent information in the gazer-head direction at the time of the observer's saccade initiations. To estimate the gazer-head angular error (relative to the gaze goal) as a function of time/video frame, a separate group of 13 research assistants (who had never seen the videos before) annotated each video frame to localize where they thought the gazer was looking at. The videos only contained the gazers with all other individuals in the videos digitally erased. This allowed us to isolate gaze direction information inherent in the gazer's head direction from peripheral information about potential gaze goals. The frames of each video were presented in a randomized order to observers to minimize sequential dependencies. The gazer vector for each frame was averaged across the 13 annotators.

### Statistical analysis

We analyzed only those saccades that were more than 2 dva in amplitude (98% of all saccades) from the initial fixation location (or the gazer location in gazer present trials). Saccades with amplitudes smaller than 2 dva were considered refixations to the gazer.

We used the non-parametric bootstrap techniques to estimate the statistical significance of the differences observed in our DVs. The bootstrap procedure accounted for observer and video variability. We resampled the 30 subject IDs with replacement and then also resampled the stimulus IDs (videos) with replacement for gazer absent vs gazer present comparisons. We then computed the mean difference in the DV per stimulus ID per subject of the bootstrap samples. For all other comparisons involving different sets of stimuli (such as between target present vs distractor present), we only resampled the 30 subject IDs with replacement and resampled the stimuli IDs with replacement separately for each condition. For each subject, we computed the mean DV for each condition across the respective bootstrapped stimulus IDs and finally computed the mean difference of the DV between the conditions being compared. All statistical analyses involved 10,000 times bootstrapped samples.

## Results

### The presence of a gazer reduces the first saccade end point error but only when the goal is far from the initial fixation location

We defined the first saccade error as the distance between the goal location and the first saccade end point. We assessed the influence of the presence of a gazer on the observers’ first saccade error (Δ first saccade error = first saccade error without gazer – first saccade error with gazer) and how it varied with the distance between the observers’ initial fixation and the goal location.

**Figure 5.**
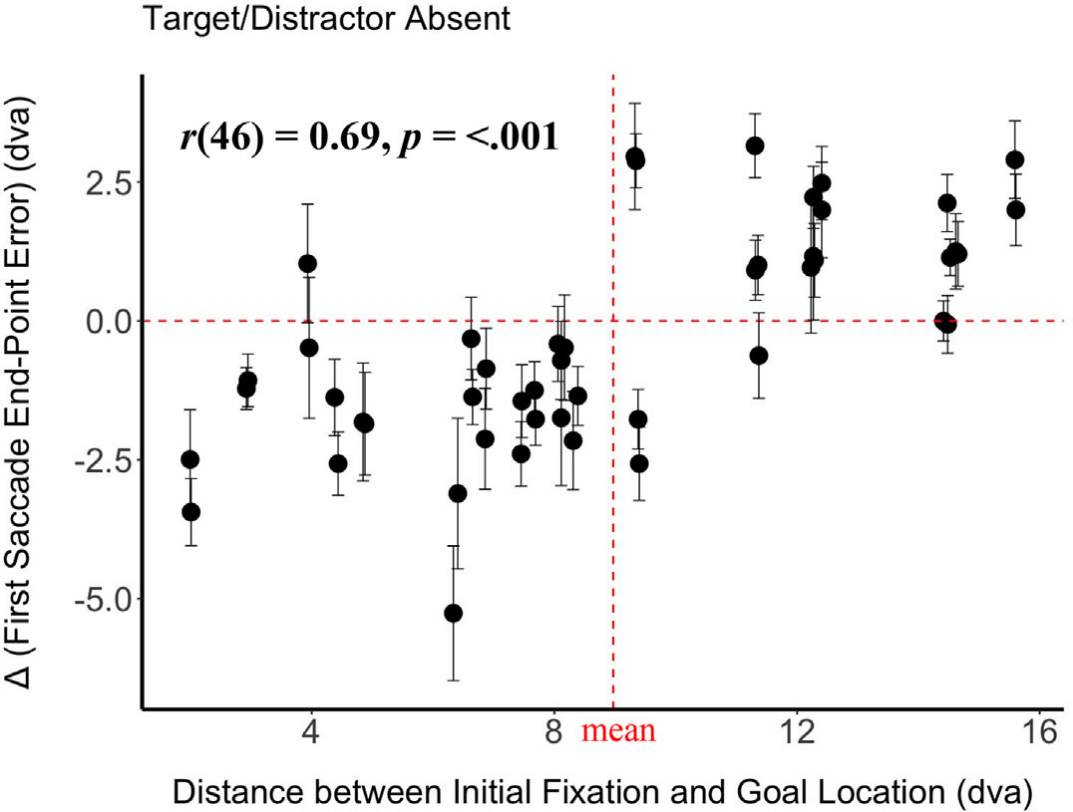
The difference in first saccade end point error between gazer absent and gazer present trials as a function of the distance from initial fixation to the goal location for goal (target/distractor) absent trials. Each point is a unique video used as a stimulus in the experiment. The error bars represent the standard error of the mean across 30 participants. Positive values of Δ indicate greater first saccade end point error when the gazer was absent, and negative values indicate greater error when the gazer was present. The vertical red dotted line indicates the mean distance between the initial fixation and goal location, while the horizontal red dotted line indicates no first saccade error difference between gazer present and absent versions of the same stimulus. Spearman correlation statistics are reported.

We observed that the benefit of the gazer's presence in reducing the first saccade error increased with the distance between the initial location and goal location, Spearman's rank correlation *r*(46) = 0.69, *p* < 0.001 (Figure 5). When the goal location was close to the initial fixation location (<9.07 dva), the first saccade error was lower when the gazer was absent vs. present. Whereas, when the initial fixation and the goal location were farther apart (≥9.07), the presence of the gazer reduced the first saccade error.

**Figure 6.**
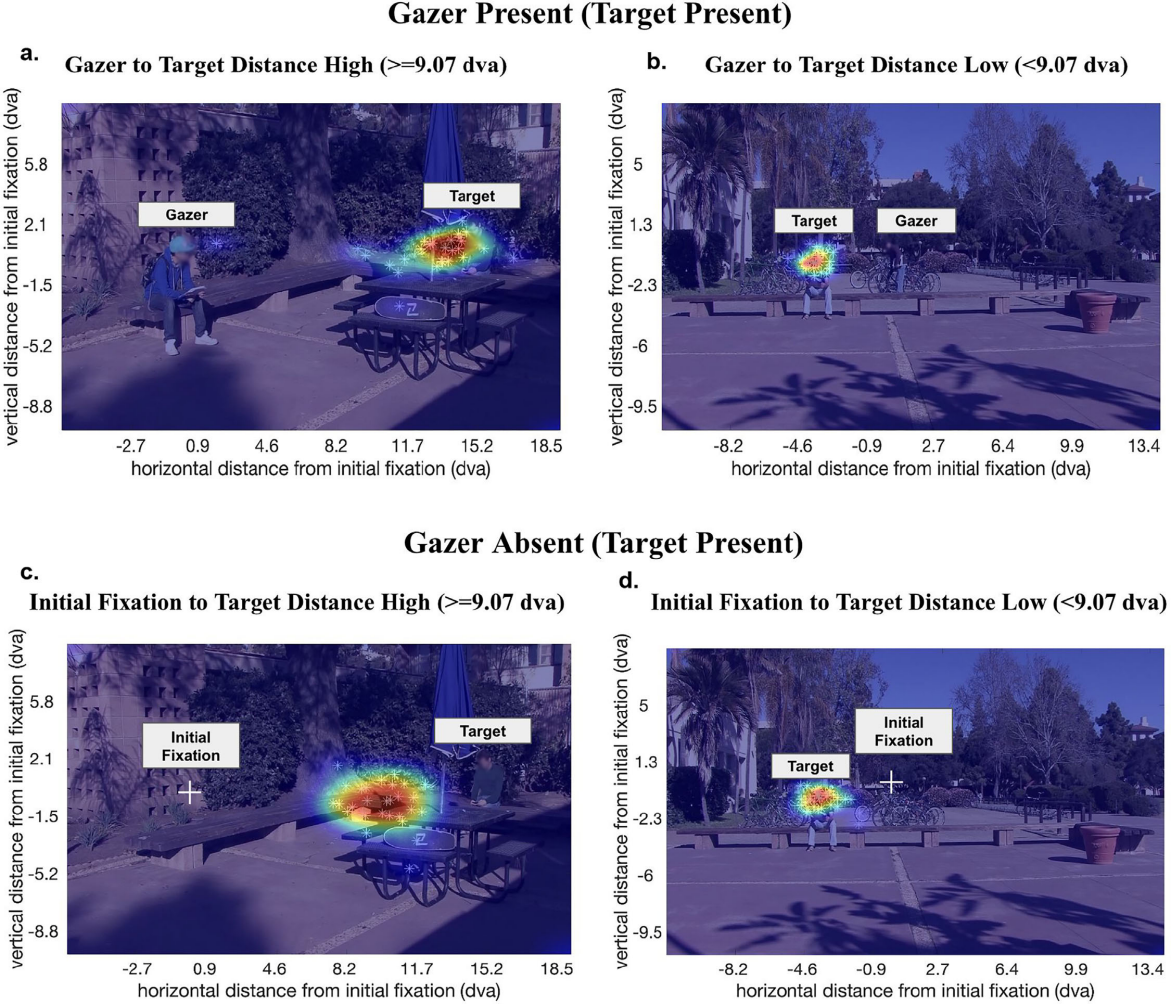
Example trials with the distribution of first saccade end points across all participants showing the presence of the gazer reduces the first saccade error only when the gaze goal (here, target) is far (≥9.07 dva) from the observer's initial fixation location: (**a**) gazer present and gazer-to-target distance is high (≥9.07 dva); (**b**) gazer present and gazer-to-target distance is low (<9.07 dva); (**c**) gazer absent and initial-fixation-to-target distance is high (≥9.07 dva); and (**d**) gazer absent and initial-fixation-to-target distance is low (<9.07 dva). Faces in the figure are blurred to protect the privacy of individuals; however, faces were displayed unaltered to the participants of the study.

Figure 6 illustrates the influence of the presence of the gazer on first saccade error by showing the heatmaps of all participants’ first saccade end points for an example trial when the goal location was far (Figures 6a and 6c) or close (Figures 6b and 6d) to the initial fixation (examples are for target-present trials; see Figure A5 for example trials in which the goal is a distractor individual).

**Figure 7.**
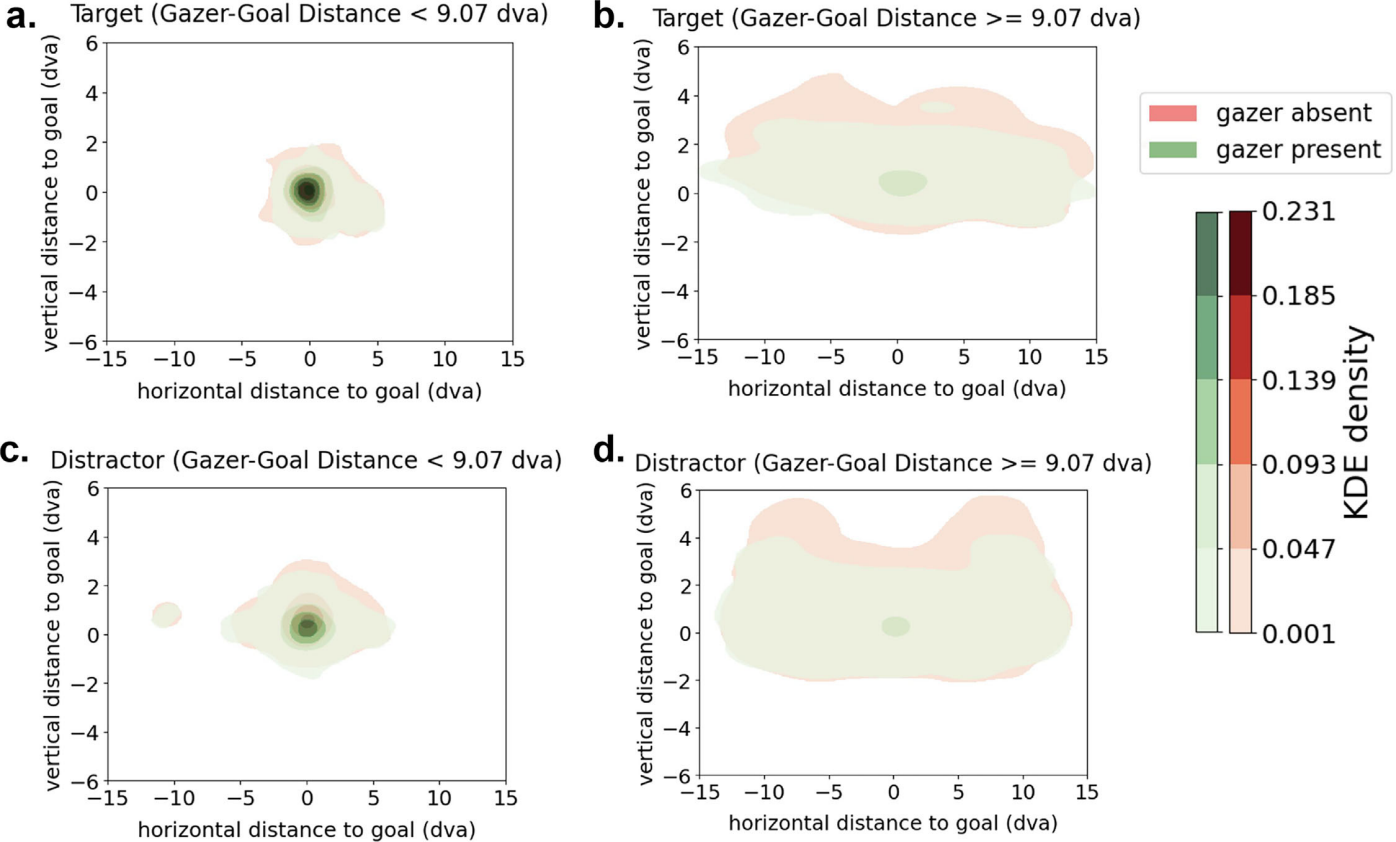
Contour maps of probability density estimates (kernel density estimations [KDEs]) of first saccade end points registered to goal locations across all trials (gazer absent vs gazer present): (**a**) goal is the target and initial-fixation-to-target distance is low; (**b**) goal is the target and the initial-fixation-to-target distance is high; (**c**) goal is a distractor and initial-fixation-to-distractor distance is low; and (**d**) goal is distractor and initial-fixation-to-distractor distance is high. The color bars indicate the estimated probability density (KDE) of all spatial locations in a given contour. A lighter shade corresponds to a lower probability density. The panels with a gazer–goal distance of ≥9.07 dva (**b** and **d**) show a lower KDE density, indicating a more diffuse spread of saccade end points distributed over a wider region.

Figure 7 shows density plots of the distribution of the first saccade end points registered to the goal location across all trials comprising different video stimuli. The same videos were presented in both gazer present and gazer absent versions and the first saccade end point distributions are shown in green and red, respectively. The spread in the distribution of the first saccade end points is higher when the goal is located more than 9.07 dva away from the initial fixation (gazer) location, irrespective of the identity of the goal as target or distractor. Further, the presence of a gazer also decreases the spread of the distribution of the first saccade end points.

**Figure 8.**
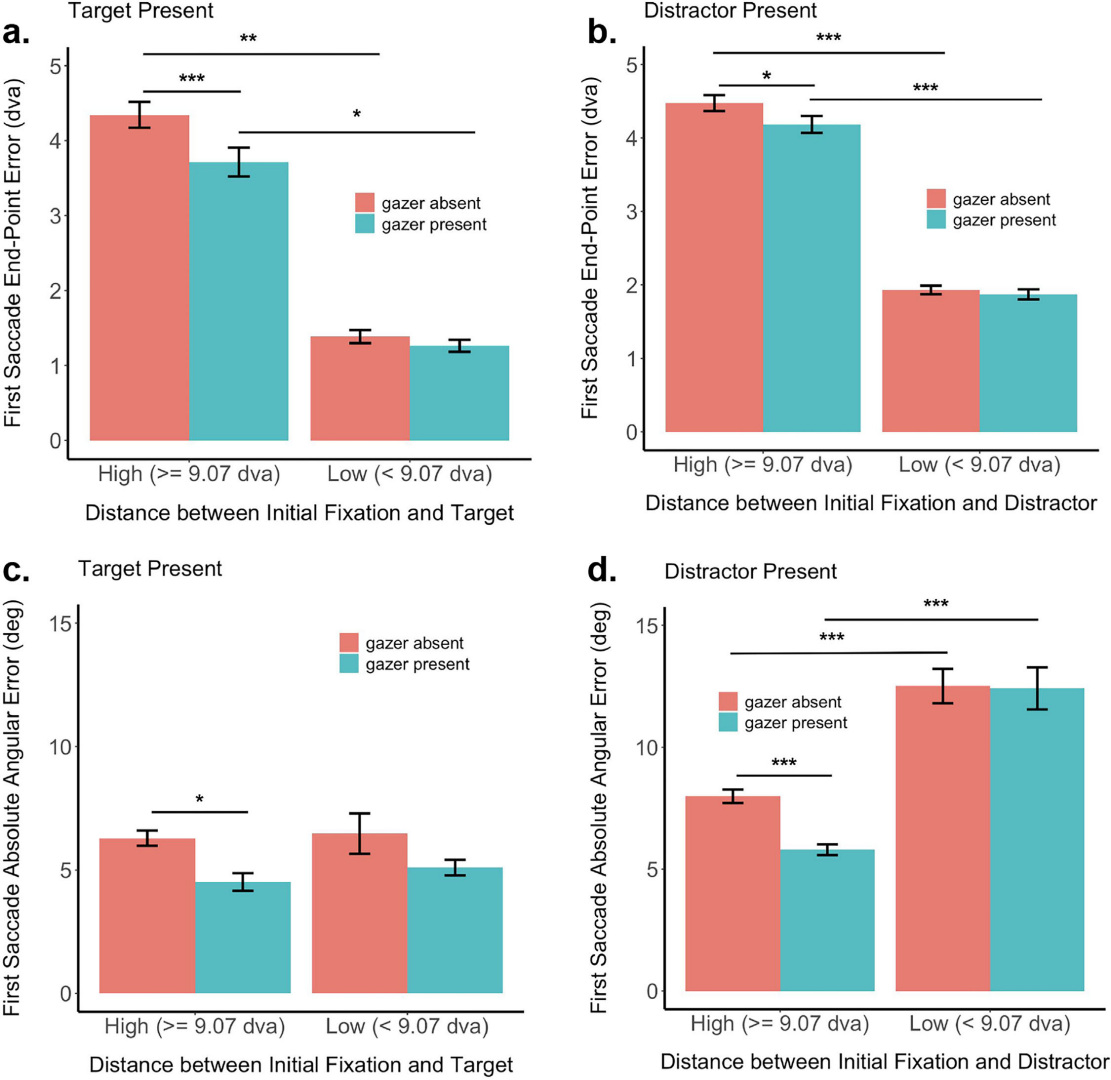
First saccade end point Euclidean and angular error. (**a**) First saccade end point Euclidean error when goal is the target. (**b**) First saccade end point Euclidean error when goal is a distractor. (**c**) First saccade end point angular error when goal is the target. (**d**) First saccade end point angular error when goal is a distractor. The error bars denote the standard error of the mean. We normalize the angular error, such that 0° indicates the true direction of the goal from the initial fixation (gazer) location, and −180°/180° indicates the opposite direction and compute the average of the magnitude of the angular error for statistical analyses.

To quantify the effect of the presence of the gazer in guiding saccade dynamics during a search task, we analyzed two measures of the first saccade for trials in which a goal was present: 1) the first saccade Euclidean error as the Euclidean distance between the first saccade end point and the true goal location (Figures 8a–b), and 2) the first saccade angular error (cosine of the angle between the vector joining the initial fixation and true goal location and the vector joining the initial fixation (goal) and the first saccade end point). We compared these measures across gaze absent and gaze present conditions.

We ran bootstrap analyses followed by false discovery rate (FDR) correction to quantify the statistical significance of any difference observed in the saccade measures between the different conditions. The first saccade end point Euclidean error was significantly higher for gazer absent than for gazer present condition both when the goal was the target (4.34 dva vs 3.71 dva, bootstrap *p* < 0.001, Cohen's d = 0.19, FDR corrected) as well as when the goal was a distractor (4.47 dva vs 4.18 dva, bootstrap *p* = 0.012, Cohen's d = 0.08, FDR corrected), but only when the initial-fixation and goal locations were at least 9.07 dva apart. A similar effect was observed when the initial fixation and goal locations were closer than 9.07 dva to each other, but the differences did not reach significance either when the goal was the target or when the goal was a distractor (Table A1). Further, the first saccade end point error was significantly higher when the initial-fixation-to-goal distance was higher than 9.07 dva, for the gazer present condition both when the goal was the target and when the goal was a distractor, as well as for the gazer absent condition both when the goal was the target and when the goal was a distractor (Figures 8a–b; Table A1).

We found similar trends in the analyses of the mean absolute angular error in the first saccade end point (see Figures 8c–d; Table A2). The first saccade end point angular error was significantly higher when the gazer was absent rather than present for both when the goal was the target (6.29° vs 4.51°, bootstrap *p* = 0.008, Cohen's d = 0.3, FDR corrected) or a distractor (7.99° vs 5.8°, bootstrap *p* < 0.001, Cohen's d = 0.28, FDR corrected), but only when the initial-fixation (gazer) and goal locations were at least 9.07 dva apart. The difference did not reach significance when the initial fixation (gazer) and goal locations were closer than 9.07 dva to each other (Table A2).

### The presence of a gazer elicits slower and higher amplitude first saccades

**Figure 9.**
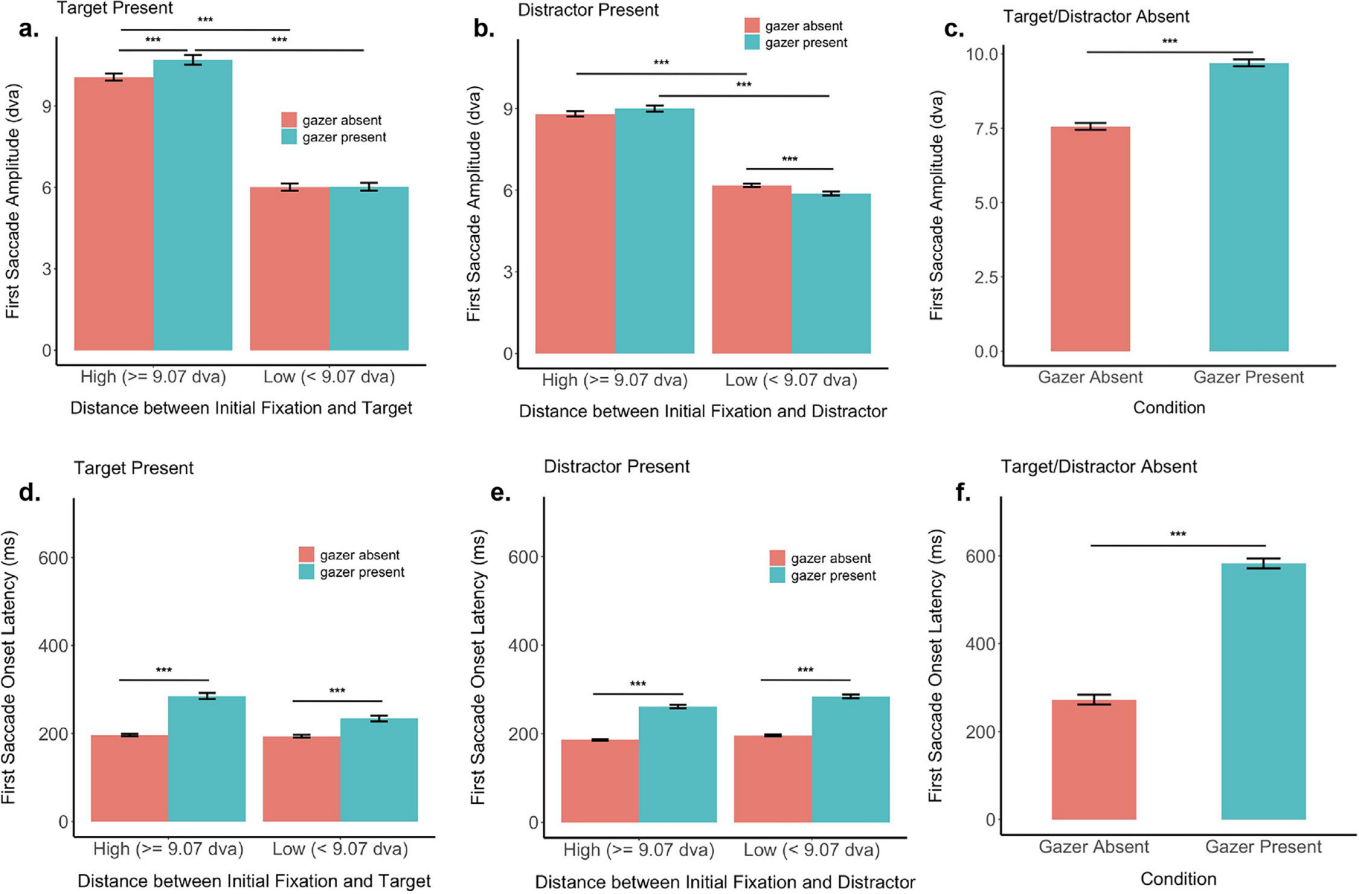
First saccade amplitude and latency. (**a**) First saccade amplitude when goal is the target. (**b**) First saccade amplitude when goal is a distractor. (**c**) First saccade amplitude when there are no goals. (**d**) First saccade onset latency when goal is the target. (**e**) First saccade onset latency when goal is a distractor. (**f**) First saccade onset latency when there are no goals. The error bars denote the standard error of the mean.

Across all conditions, including those in which no goal was present, we compare two more measures of the first saccade between gazer absent and gazer present conditions: 1) the first saccade amplitude, defined as the distance between the initial fixation (gazer) and the first saccade end point (Figures 9a–c), and 2) the first saccade onset latency, defined as the time elapsed between the start of the trial and the onset of the first saccade (Figures 9d–f).

First, for both gazer absent and gazer present conditions, irrespective of whether the goal was the target or a distractor, the first saccade amplitude was significantly larger when the goal was located farther away from the initial fixation location (Figures 9a–c; Table A3).

Next, we found that when the goal was the target located at least 9.07 dva away from the initial fixation (gazer) location, the first saccade amplitude was significantly larger when the gazer was present than absent (10.7 dva vs 10.07 dva, bootstrap *p* < 0.001, Cohen's d = 0.23, FDR corrected). The same was observed when there were no goals (9.7 dva vs 7.56 dva, bootstrap *p* < 0.001, Cohen's d = 0.62, FDR-corrected). The influence of the gazer on saccade amplitude did not reach significance when the goal was a distractor located more than 9.07 dva away from initial fixation (Table A3).

Finally, when the goal was the target located closer (<9.07 dva) to the initial fixation (gazer) location, the first saccade amplitude was not significantly different between the gazer present and absent conditions. However, when the goal was a distractor located closer to the initial fixation, the first saccade amplitude for the gazer absent condition was significantly larger than the gazer present condition (Table A3). This indicates that when the distractor was closer in their visual periphery, and no foveal gazer was present, observers were able to ignore the closer distractor by making larger saccades. This effect was eliminated when a foveal gazer was present, guiding observers’ saccades correctly to the closer goal distractor. This is also reflected in higher first saccade errors when the goal was a distractor located close to the initial fixation location, and a foveal gazer was absent (Table A6).

The presence of the gazer increased the first saccade onset latency across all conditions (Figures 9d–f; Table A4). In addition, the first saccade onset latencies were significantly longer when no peripheral goals were present than when one or more goals were present (for both gazer present and gazer absent conditions (Table A4).

The findings are consistent with the interpretations that the presence of the dynamic gazer at the initial fixation location requires that observers take additional time to process the foveal visual information resulting in increased first saccade onset latencies.

### The presence of two potential goals in the periphery elicits larger error in the first saccade

**Figure 10.**
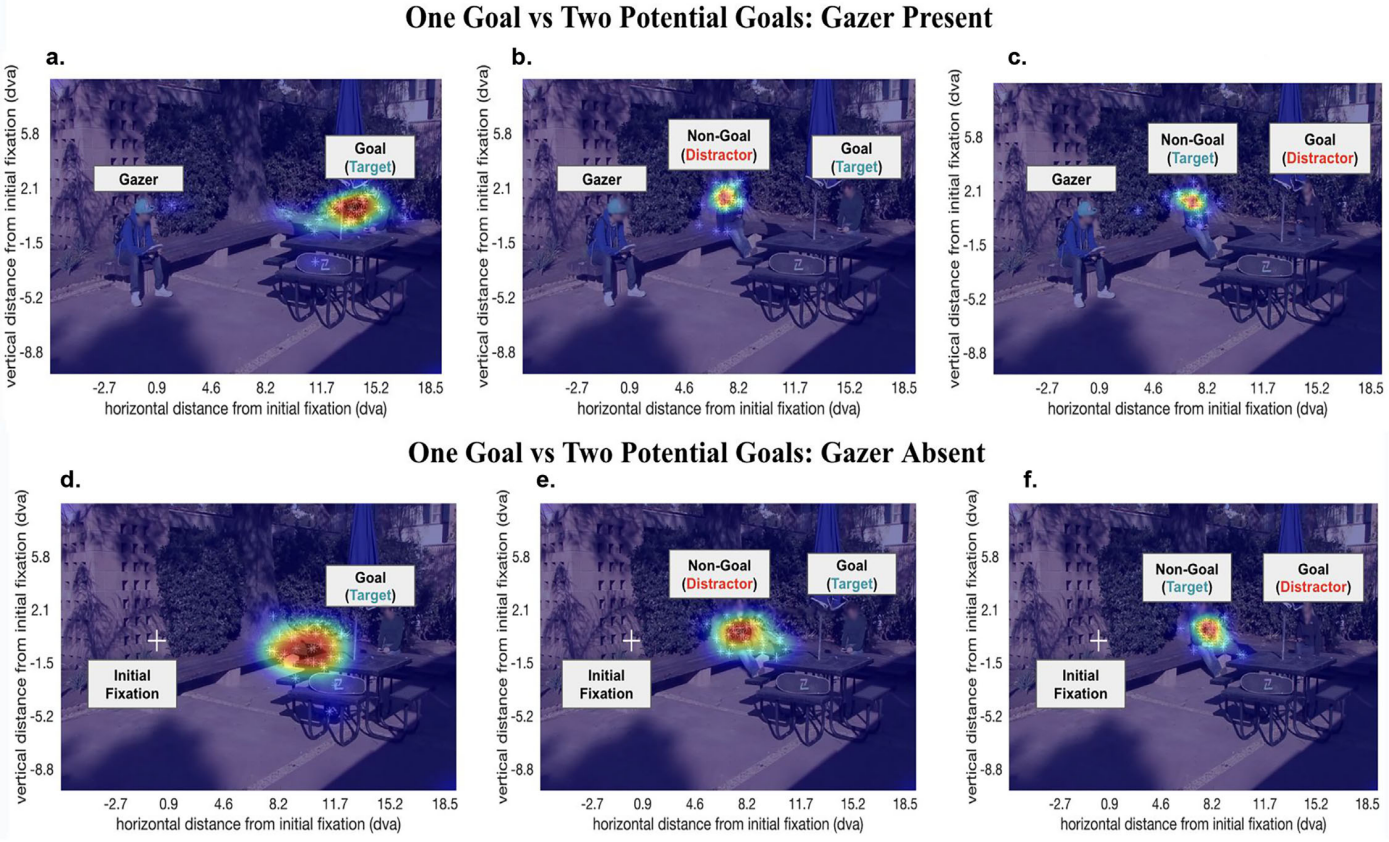
Observers tend to look at the closer individual first when two potential gaze goals are present. Example trials with the distribution of first saccade end points across all participants: (**a**) gazer is present with one goal; (**b**) gazer is present with two potential goals and the goal is located farther from the gazer than the non-goal; (**c**) gazer is present with two potential goals, and the goal is located closer to the gazer than the non-goal; (**d**) gazer is absent with one goal; (**e**) gazer is absent with two potential goals and the goal is located farther from the gazer than the non-goal; and (**f**) gazer is absent with two potential goals and the goal is located closer to the gazer than the non-goal. Faces in the figure are blurred to protect the privacy of individuals; however, faces were displayed unaltered to the participants of the study.

We had included the two potential goals condition to emulate real-world scenarios more closely: it rarely happens that we search for one specific person in an otherwise empty background. We included the separate sub-conditions, one goal and two potential goals, to explore how gaze-following saccade dynamics are affected by added uncertainty in the search task. Figure 10 shows the heat maps of the distribution of the first saccade end points of all participants in specific example trials. For the two potential goals condition, we created pairs of videos where the target and a distractor were spatially swapped. Thus, in one video the target person was the gaze goal while the distractor was the non-gaze goal, and in the corresponding video with the digital swap, the distractor was the gaze goal and the target was the non-gaze goal. This digital manipulation allowed us to control for all other visual information in the videos. For instance, Figures 10b and 10c, as well as Figures 10e and 10f, are such stimulus pairs. Our findings show that observers fixated first on the individual closer to the initial fixation (gazer) location irrespective of whether that person is the goal or the target, and irrespective of whether a gazer is absent or present. This is analyzed in further detail in the next subsection.

**Figure 11.**
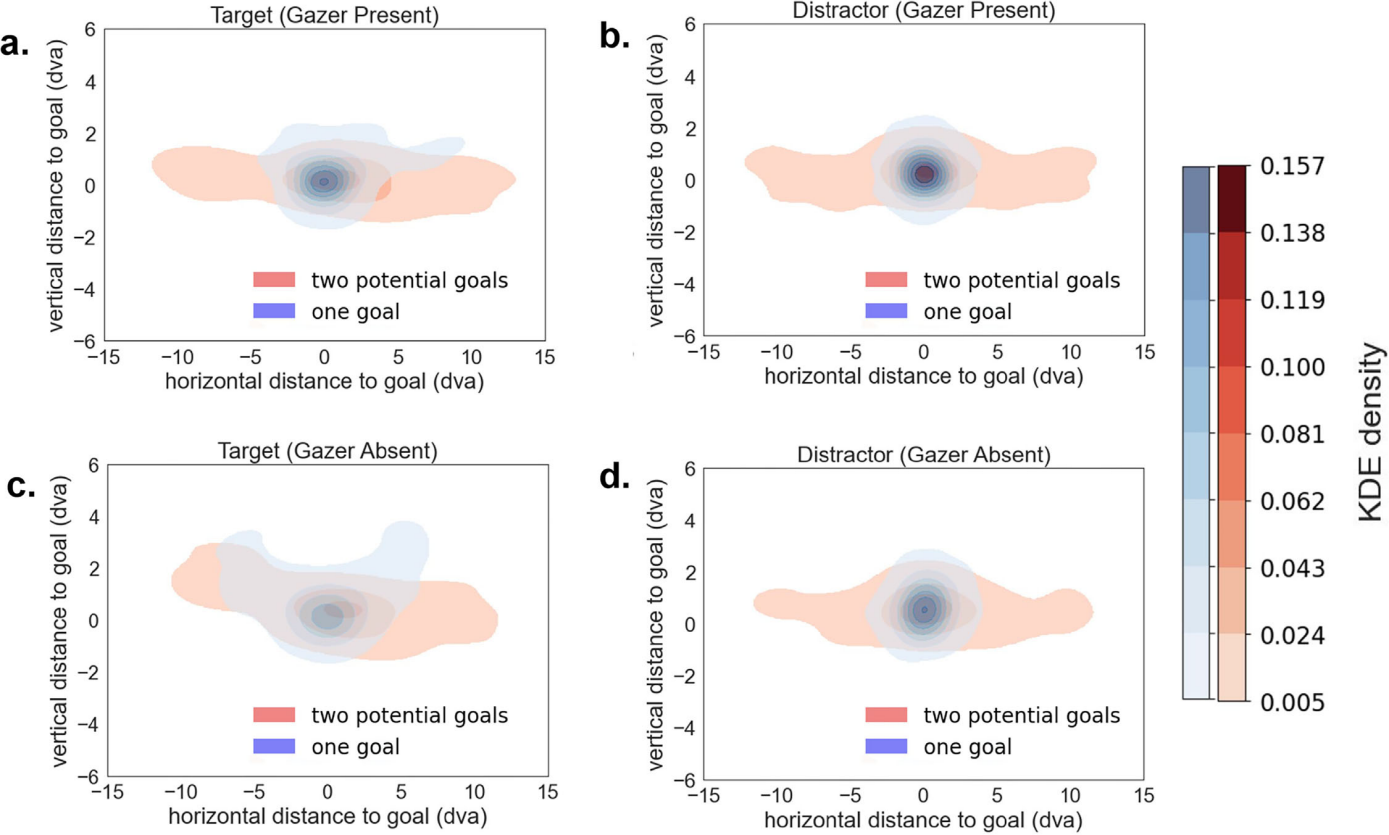
Contour maps of probability density estimates (kernel density estimations [KDEs]) of first saccade end points registered to goal locations across all trials (one goal vs two potential goals): (**a**) gazer present with the target as the goal; (**b**) gazer present with a distractor as the goal; (**c**) gazer absent with the target as the goal; and (**d**) gazer is absent with a distractor as the goal. The color bars indicate the estimated probability density (KDE) of all spatial locations in a given contour. A lighter shade corresponds with a lower probability density.

Figure 11 shows density plots of the distribution of the first saccade end points registered to the goal locations separated by the number of potential goals present, the presence of the gazer, and the type of the goal (target/distractor). Irrespective of whether the gazer is absent or present and whether the goal is the target or a distractor, the first saccade end points have a larger spread in the presence of two potential goals than when only one goal is present.

**Figure 12.**
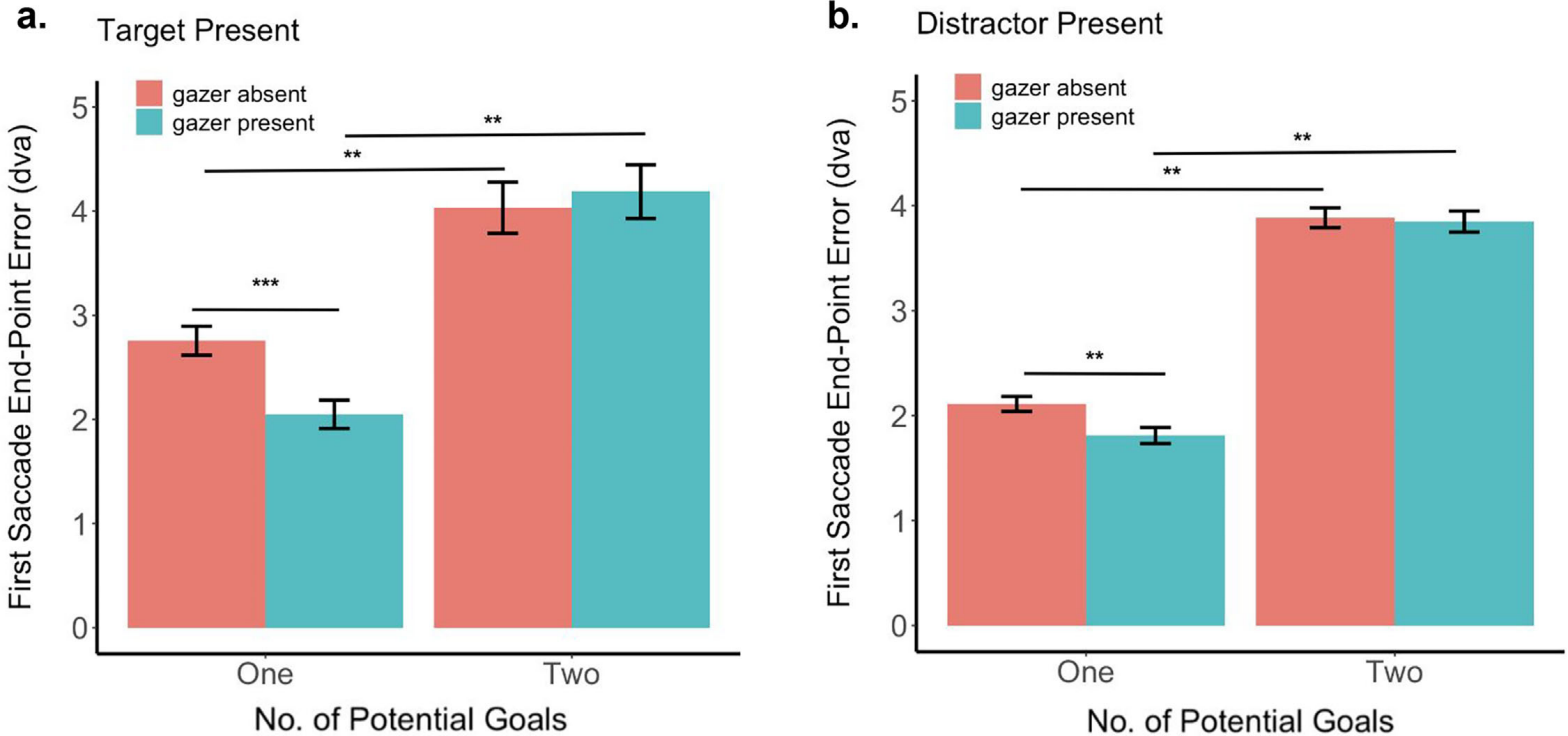
First saccade end point Euclidean error. (**a**) Goal is the target. (**b**) Goal is a distractor. The error bars denote the standard error of the mean.

To quantify the effect of multiple potential goals in the search task, we compared the first saccade end point Euclidean error between one and two potential goals, as well as between the gazer absent and gazer present conditions. We found that the presence of two potential goals elicits a significantly larger error in the first saccade as compared to when only one goal was present, irrespective of whether a gazer was present and whether the goal was a target or a distractor (Figure 12; Table A5).

In addition, we found that when only one goal (target or distractor) is present, the presence of the gazer reduces the first saccade error (goal target: 2.75 dva vs 2.05 dva, bootstrap *p* < 0.001, Cohen's d = 0.27 FDR corrected; goal distractor: 2.11 dva vs 1.81 dva, bootstrap *p* = 0.009, Cohen's d = 0.13, FDR corrected). This suggests that, when peripheral information is not uncertain, the gazer's benefit in guiding the first saccade is more evident. To understand why this might be the case, we analyzed the saccade data for conditions in which two potential gaze goals were present, as discussed elsewhere in this article.

### The first saccade lands at the individual closer to the initial fixation when two potential goals are present in the direction of gaze

**Figure 13.**
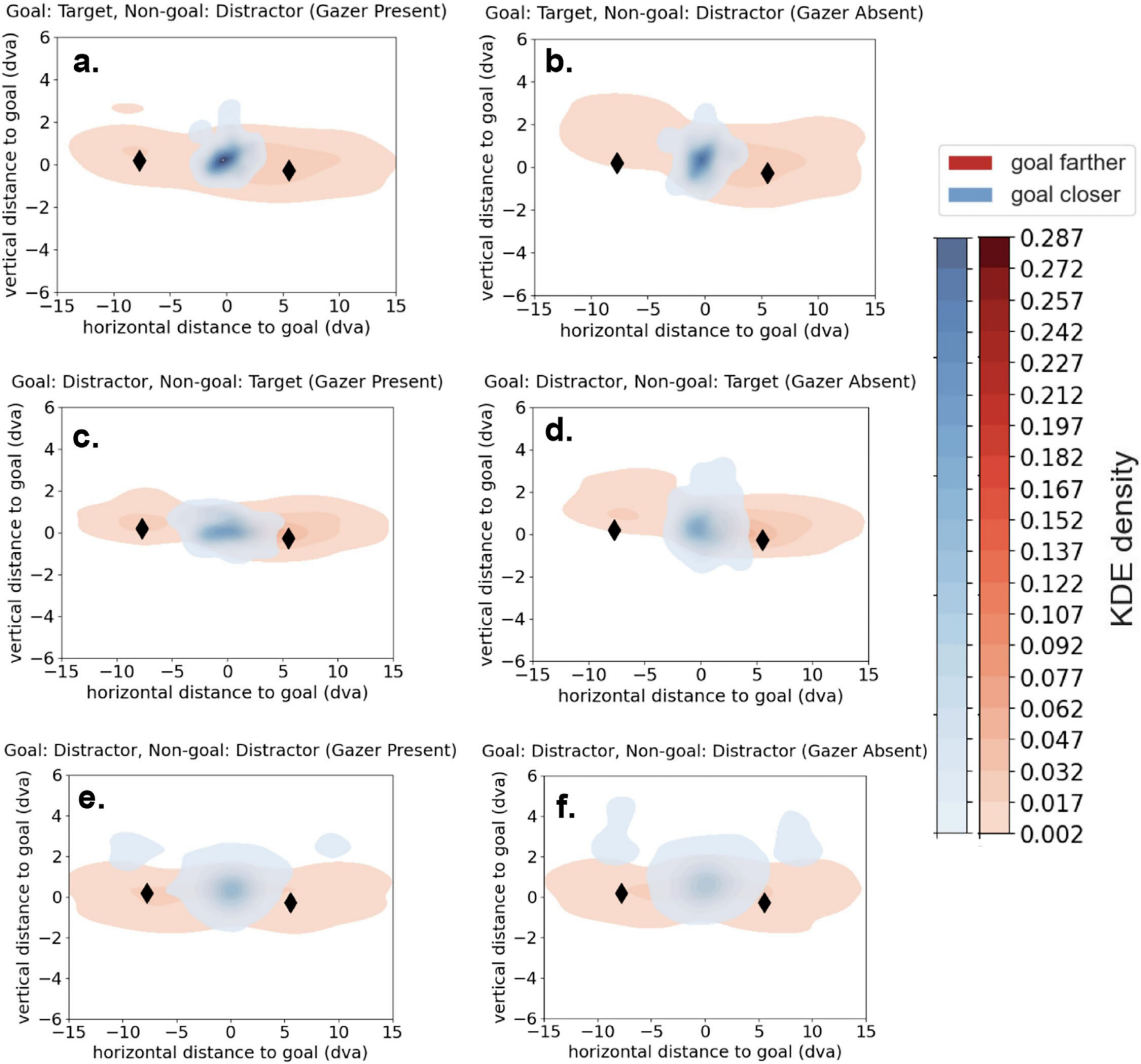
Contour maps of probability density estimates (kernel density estimations [KDEs]) of first saccade end points registered to goal locations across all trials consisting of two potential goals (goal Closer vs goal Farther): (**a**) gazer is present, goal is the target, and non-goal is a distractor; (**b**) gazer is absent, goal is the target, and non-goal is a distractor; (**c**) gazer is present, goal is a distractor, and non-goal is the target; (**d**) gazer is absent, goal is a distractor, and non-goal is the target; (**e**) gazer is present, goal is a distractor, and non-goal is a distractor; and (**f**) gazer is absent, goal is a distractor, non-goal is a distractor. The black diamonds indicate the average location of the non-goal to the left and right of the goal location in trials where the non-goal is closer to the initial fixation (gazer) location. The color bars indicate the estimated probability density (KDE) of all spatial locations in a given contour. A lighter shade corresponds with a lower probability density.

Next, we assessed the first saccade end point when two potential goals were present in the trials. Figures 10b and 10c and Figures 10e and 10f show that when there are two potential goals, the observer's first saccade usually lands at the individual closer to the initial fixation (gazer) location regardless of whether that individual is the target, a distractor, the goal or the non-goal in the given scene. Figure 13 shows density plots of the distribution of the first saccade end points registered to the goal locations for trials with two potential goals, wherein the goal was either closer or farther than the non-goal to the gazer. Across all conditions, the first saccade end points have a larger spread when the goal is farther than when it is closer. Additionally, when the goal is farther, the first saccade end point distribution shows two modes, near the location of the non-goal that is closer to the initial fixation (gazer) location either to the left or right of the goal location (as indicated by black diamonds in Figure 13).

**Figure 14.**
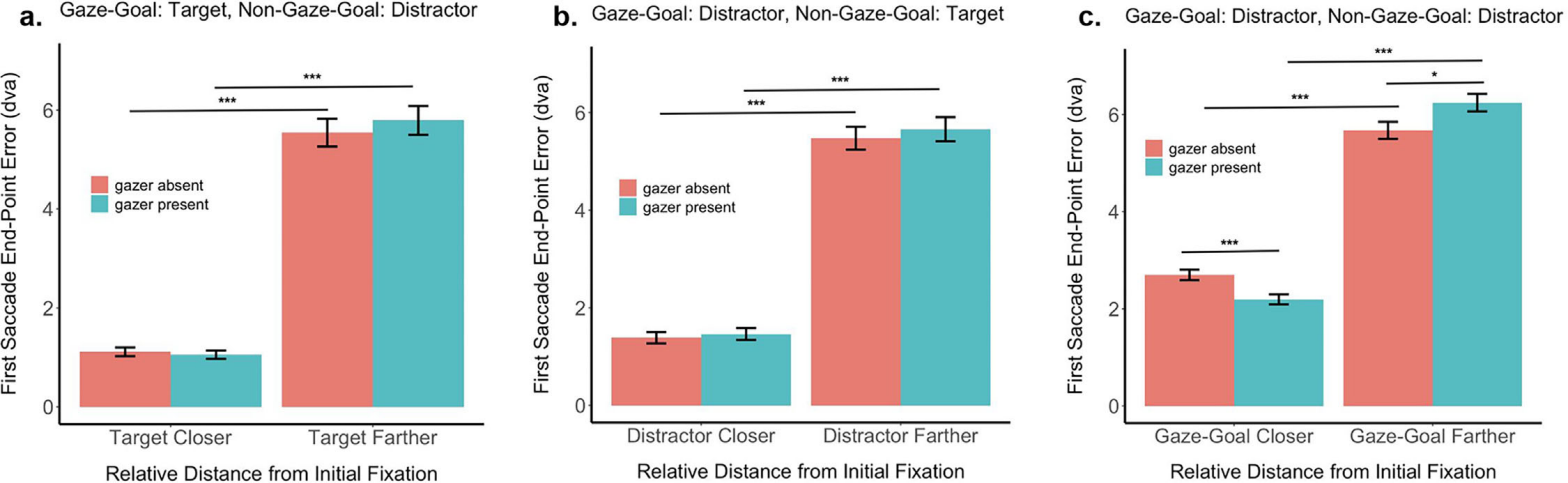
First saccade end point error (goal closer vs goal farther): (**a**) goal is the target and non-goal is a distractor; (**b**) goal is a distractor and non-goal is the target; and (**c**) both goal and non-goal are distractors. The error bars denote the standard error of the mean.

To further quantify the observed differences in the first saccade end point distributions between trials in which the goal is closer and those in which the goal is farther, we analyzed the first saccade end point Euclidean error. The first saccade Euclidean error is significantly larger when the goal is farther from and the non-goal is closer to the initial fixation (gazer) location, regardless of the identity of the goal and the non-goal and whether the gazer is present or not (Figure 14; Table A6).

In addition, for the trials in which both goal and non-goal were distractors, and the goal distractor was closer to the initial fixation location than the non-goal distractor, the first saccade end point error for the gazer absent condition is significantly larger than the gazer present condition (2.7 dva vs 2.19 dva, bootstrap *p* < 0.001, Cohen's d = 0.19, FDR corrected). This finding is in line with the hypothesis that observers ignore the peripherally closer distractor while searching for the peripheral target, in the absence of a foveal gazer (Table A3). This increases the first saccade error when the goal is a distractor located close to the initial fixation location.

Following this logic, if the gazer is absent, and if the goal is a distractor located far from the initial fixation location while the closer non-goal is also a distractor, observers would similarly ignore the peripherally closer distractor to fixate the farther distractor first (who is now the goal). This ability to ignore the closer distractor would be diminished in the presence of a foveal gaze cue. Thus, we expect the first saccade error to be lower in the absence of a foveal gazer for this case. And this is exactly what we observe: the error for the gazer present condition is significantly larger than the gazer absent condition when the goal is farther from the initial fixation location than the non-goal (6.25 dva vs 5.68 dva, bootstrap *p* = 0.012, Cohen's d = 0.16, FDR corrected).

### One goal vs two potential goals: Selection vs precision error

**Figure 15.**
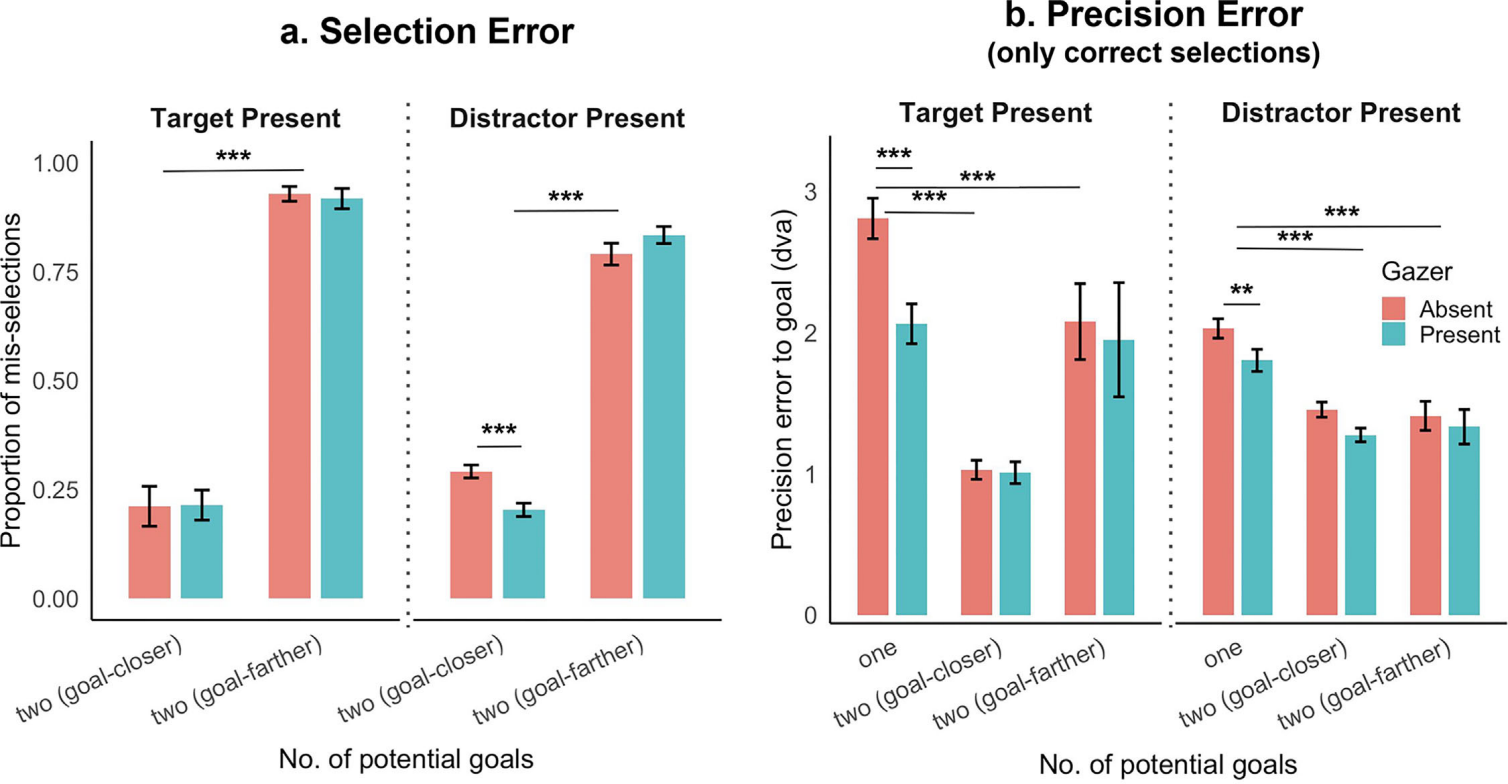
One goal vs two potential goals, Selection error (**a**) and precision error (**b**). (**a**) Proportion of all two potential goals trials, split by gazer present vs absent, goal closer vs farther, and target vs distractor present, that suffered from mis-selection (first saccade landed closer to non-goal than to the goal). (**b**) Precision error to goal for all correct selections (first saccade landed closer to goal than to non-goal) and presents the one goal condition for comparison. In general, the trends from our first saccade Euclidean error analysis are reflected here: the presence of a gazer reduces first saccade error for the one-goal condition, and a first saccade error selection error is lower for closer goals than farther goals when more two potential goals are present. Additionally, in target absent trials, correct selections have similar precision errors between trials with closer vs farther goals.

So far, we have reported the Euclidean distance between the first saccade end point and the goal location as our main measure of the first saccade error. However, when there are two potential goals, the first saccade end point may result in a selection error: when the first saccade lands closer to the non-goal that the goal, or a precision error: wherein the first saccade lands closer to the goal but not exactly at the goal location. Our Euclidean distance analysis between the first saccade end point and the goal location includes both the selection and the precision error. To separate the two kinds of errors (selection and precision) and to compare the precision for only correct selections between the two potential goals and one goal conditions, we ran the following analysis. First, for each trial and subject, we computed the Euclidean distance between the first saccade end point and the goal location (distance *m*), and that between the first saccade end point and the non-goal location (distance *n*). Next, for the trials with two potential goals, we classify trials with distance *n* < distance *m* as mis-selections (selection error) (Figure 15a). Finally, we report the precision error (Euclidean distance between first saccade end point and goal location) for all trials with correct selections (distance *m* < distance *n*) and present the one-goal condition for comparison (Figure 15b). We observe that the trends from our first saccade Euclidean error results are reflected in this analysis. First, the proportion of mis-selection of the non-goal is significantly higher when the goal is farther than closer in the two potential goals condition. Second, in target absent trials, the gazer reduced the proportion of mis-selections in goal-closer trials. Third, for trials in which the first saccade correctly selected the goal, the presence of a gazer significantly reduced precision error for one-goal but not the two potential goals condition, aligning with our finding in Figure 12.

### Observer's first saccades anticipate the gazer's head direction

**Figure 16.**
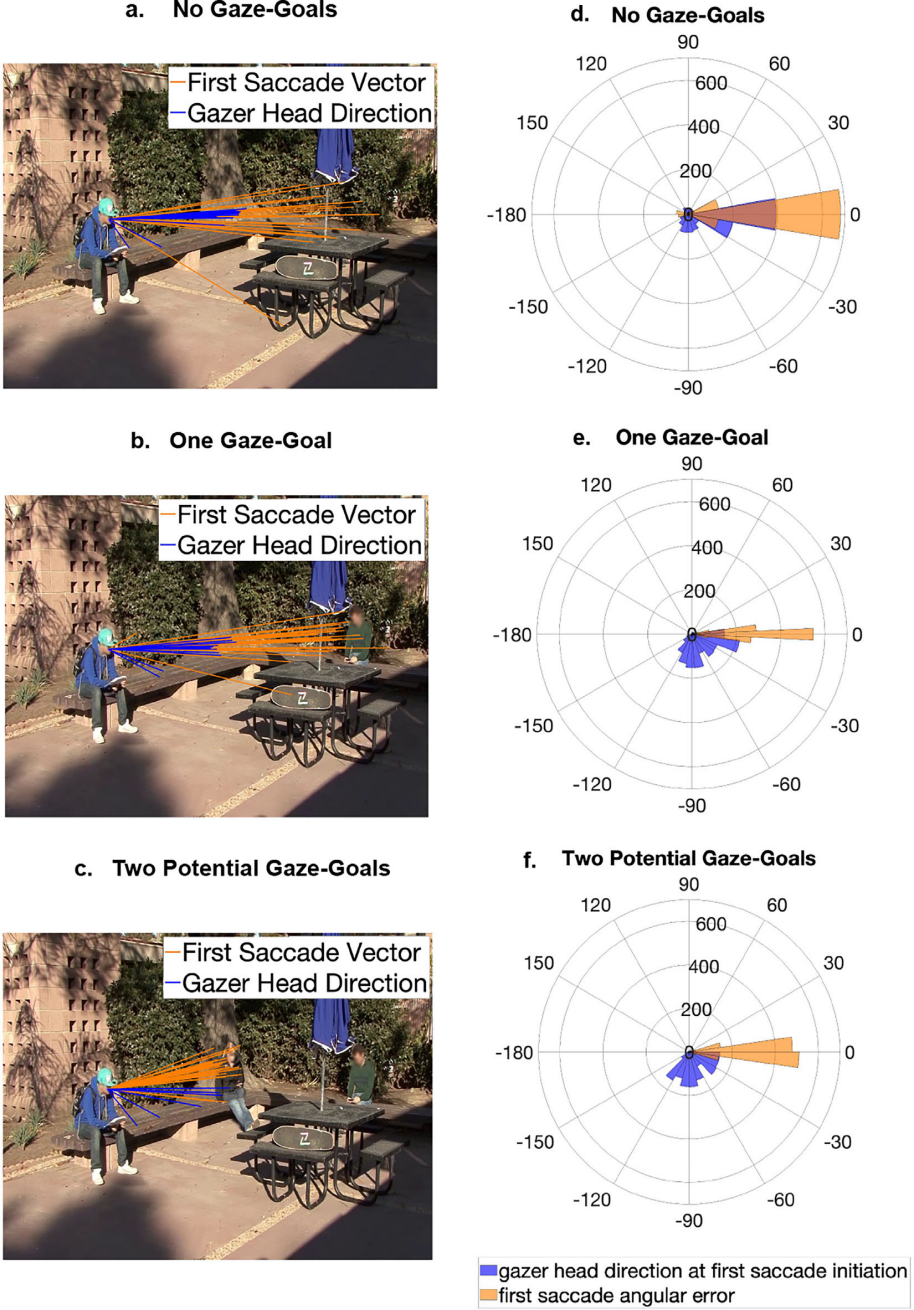
Observers' first saccades anticipate dynamic gaze cues. (**a**) No gaze goals, example trial. (**b**) One gaze goal, example trial. (**c**) Two potential gaze goals, example trial. (**d**) No gaze goal, registered across all trials. (**e**) One gaze goal, registered across all trials. (**f**) Two potential gaze goals, registered across all trials. The blue lines show the direction of gazer head at the time of first saccade initiation for each observer and the orange lines depict first saccade direction for each observer. In (**d**–**f**), the 0° line indicates the final gazer-head direction at the time of looking at the gaze goals, registered across all trials. Faces in the figure are blurred to protect the privacy of individuals; however, faces were displayed unaltered to the participants of the study. at the time of first saccade initiation as a measure of anticipation of gaze direction.

For this analysis, we used only those trials in which a gazer was present. Using the annotations made by 13 research assistants who did not participate in the study in a procedure described in the methods section, we estimated the gazer's head direction at the time of initiation of the first saccade. Figures 16a–c show the estimated gazer's head direction (blue lines) at the time of initiation of the first saccade whereas the orange lines are the first saccade vectors. Figures 16d to 16f show the data for all trials using polar histograms registered relative to the final gazer's head direction angle (labeled as 0). The gaze end point location estimated by the 13 annotators varied with an average standard error of 0.9 dva in the *x* direction and 0.3 dva in the *y* direction.

We found that in the absence of any gaze goal, the mean (across images and observers) gazer head direction at the time of the first saccade initiation was 48.63° away from the gaze goal direction. When only one gaze goal is present, the mean gazer head direction at the time of the first saccade initiation was 62.62°. Finally, when two potential gaze goals are present, the mean gazer head direction at the time of the first saccade initiation was 69.11°. Furthermore, the observers’ first saccades anticipated the gazer's head movement by 100 ms in the absence of any gaze goals, by 394 ms in the presence of one gaze goal, and by 426 ms in the presence of two potential gaze goals. While the anticipation is much higher in the presence of one or more individuals in the periphery, bootstrap statistics show that the anticipation of the gazer's head direction is significantly larger when two potential gaze goals are present than when no gaze goals are present (426 ms vs 100 ms, bootstrap *p* = 0.04, Cohen's d = 0.45) (Table A7).

## Discussion

Our study aimed to extend previous research assessing the dynamics of gaze following eye movements to more realistic settings that included multiple potential gaze goals in natural scenes. We analyzed observers' eye movements as they completed a dynamic visual search task in the presence or absence of gaze cues. We investigated the effects of various interacting elements of a naturalistic scene: the presence of a foveal gazer, the presence of one or more peripheral individuals as potential gaze goals, and the relative spatial location of these individuals. These insights can inform theories of social attention and real-world decision-making, offering a richer understanding of how we navigate complex social scenes.

### Foveal gazer increases observers’ first saccade latency and amplitude

The latency of the first saccade is defined as the time duration between stimulus onset and the start of the first saccade. Saccades are essentially sensorimotor decisions, wherein the visual system must use available foveal and peripheral information to guide the upcoming gaze shift to a new location for the next fixation (Nuthmann, Smith, Engbert, & Henderson, 2010; Tatler, Brockmole, & Carpenter, 2017; see Glimcher, 2003 for a review). Saccade planning involves foveal processing and peripheral selection in parallel (Ludwig, Davies, & Eckstein, 2014; but see Wolf, Belopolsky, & Lappe, 2022). Thus, the latency of a saccade reflects the time required for foveal and peripheral processing during saccade planning. We show that the first saccade latency was longer when a dynamic foveal gazer was present than absent, irrespective of the presence or absence of peripheral goal information. This finding suggests that the shorter saccade latencies in the absence of a foveal gazer may be attributed to the absence of any meaningful information in the observer's fovea at the start of a trial. This is also consistent with previous studies reporting the gap effect: wherein the removal of a central fixation point before the onset of a peripheral target significantly reduces saccade latencies compared to when the central fixation point remains during the whole trial (Pratt, Lajonchere, & Abrams, 2006; Saslow, 1967).

In addition, longer latencies may reflect either increased foveal processing, or increased peripheral processing, or both. Our data shows that in the absence of any peripheral information (no goal condition), first saccade latencies were longer and amplitudes larger when a foveal gazer was present (Figures 9c and 9f). This led to increased first saccade end point error if the goal location was close and decreased error if the goal location was far (Figure 5). Further, there was an overall reduction in saccade latencies when peripheral goals were present in addition to a foveal gazer (Figures 9d, 9e), compared to the gazer only (no goal) condition. Yet, even here, first saccade latencies were longer when a foveal gazer was present vs absent. Taken together, our data show evidence for a stronger influence of foveal (gazer) processing, rather than peripheral processing, on saccade latency during dynamic gaze following.

The presence of a foveal gazer also led to an increased first saccade amplitude compared to gazer absence, especially in the absence of any peripheral (goal or non-goal) information. The amplitude of the first saccade was reduced when there were individuals present as goals or non-goals, indicating that peripheral information influences saccade landing location. One plausible explanation behind the increased saccade amplitude in the presence of a gazer and especially in the absence of a goal may relate to the gaze direction of the gazer. The gazers in our videos usually looked horizontally toward the left or the right, rather than gazing vertically upwards or downwards, thus allowing for a greater range of likely locations along the horizontal axis that influenced larger saccade amplitudes.

### Foveal gazer guides observers’ first saccade when the peripheral goal is far from initial fixation

Our experiment was designed to enforce the observer's initial fixation at the gazer's head (or the same location in the background for gazer-absent trials) when the trial starts. We found that the presence of a gazer reduces first saccade end point error in locating the goal, but only when the gazer and the goal are located far from each other. Further, we found that the first saccade onset latency is higher in the gazer present condition. This is evident because observers spend more time processing the information in the gazer's head, which is then used to guide subsequent saccades. Moreover, the amplitude of the first saccade is also affected by the gazer: when a gazer is present, the first saccade amplitude is significantly larger than when a gazer is absent. In other words, observers make longer saccades when foveal gazer information is available. On the contrary, observers’ shorter first saccades in the absence of the gazer result in higher saccade end point errors, especially when the initial fixation and goal locations are farther apart. The greater saccade amplitudes in the presence of the gazer might reflect a saccade planning strategy that is based on prior knowledge of distances between the gazer and gaze goals in real scenes.

Further, we show that having an individual visible in the periphery already guides the observer's saccade toward that individual, irrespective of a foveal gazer being present or not (Figure 8). Indeed, there is an interplay between foveal gazer and peripheral goal information, and in some cases (less than approximately 9 dva between gazer and goal), the foveal gazer information is not as important as the peripheral goal. In this case, the goal is resolved enough in the periphery to guide the observer's saccade accurately. However, beyond this, when the gazer and goal are located farther away (more than approximately 9 dva) from each other, we observe a significant beneficial effect of the gazer's head direction in reducing both Euclidean distance error as well as angular error in observers’ first saccades (Figure 8).

Gaze cues have been known to be powerful in orienting attention toward the gazed direction (Driver et al., 1999; Friesen et al., 2004). Our study adds to existing knowledge by showing that observers plan slower and longer saccades during search when gazer information is available and use this information to guide saccades more efficiently toward the gazed location. This effect is more pronounced when the gazer and the gazed location are situated farther apart. In these cases, the peripheral gaze goal information is degraded (van Beers, 2007; Koehler et al., 2017b), and here, the presence of the gazers reduced the first saccade end point error. However, when the initial fixation and goal location are near each other, observers can rely heavily on the more accessible peripheral information to program the saccade end point location. Thus, in this case, the presence of the gazer has less impact on guiding the eye movements. Together, these findings provide an example of real-world circumstances for which the human brain integrates foveal and peripheral information (Kroell & Rolfs, 2022; Liang, Maher, & Zhaoping, 2023; Ludwig et al., 2014; see Stewart, Valsecchi, & Schütz, 2020, for a review) during visual search.

### Uncertainty about goal location increases first saccade end point error

**Figure A2.**
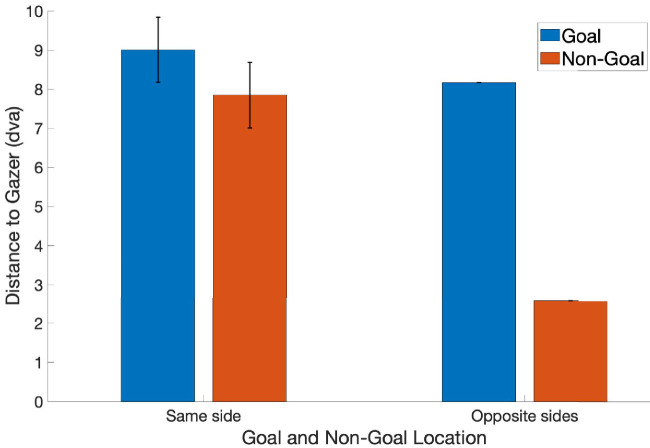
Average distance of the goal and the non-goal individuals from the gazer. Of our 24 unique video stimuli, 23 videos had the goal and the non-goal located on the same side of the gazer, although 1 video had the goal and the non-goal on opposite sides of the gazer. The same side vs opposite side classification was done based on the two-dimensional horizontal locations of gazer, goal and non-goal. Importantly, within the same side videos, the distance of the gazer to the goal and to the non-goal were not significantly different from each other, t(22) = 0.995, *p* = 0.35, 95% CI = −1.168 to 3.569. In the one video where the goal and the non-goal were on opposite sides of the gazer, the goal was located farther (approximately 8 dva) from the gazer than the non-goal (approximately 2.5 dva). Of the 24 videos, 11 had the goal closer to the gazer than the non-goal, whereas the remaining 13 had the non-goal closer to the gazer than the goal. The goal (or non-goal) could either be the search target person (25%) or a distractor person.

**Figure A3.**
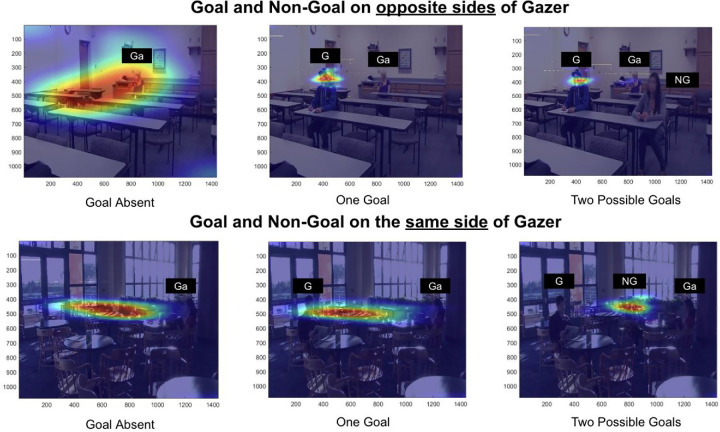
Heatmaps of first saccade end points on example trials with goal and non-goal on opposite sides vs on the same side of the gazer. (Top) A frame from one of the opposite sides video. (Bottom) A frame from 1 of the 23 same side videos. (Left) First saccade end point heatmaps when no goals were present (only gazer present). (Middle) One goal present trials. (Right) Two potential goals present trials. Importantly, in the opposite sides video, the first saccades end at the goal location even when a closer non-goal is present. Whereas the same side example shows our general finding: observers fixate the closer individual first, regardless of goal or non-goal, when there are two potential goals present in the direction of gaze. *Notes*: G, goal; Ga, gazer; NG, non-goal.

**Figure A5.**
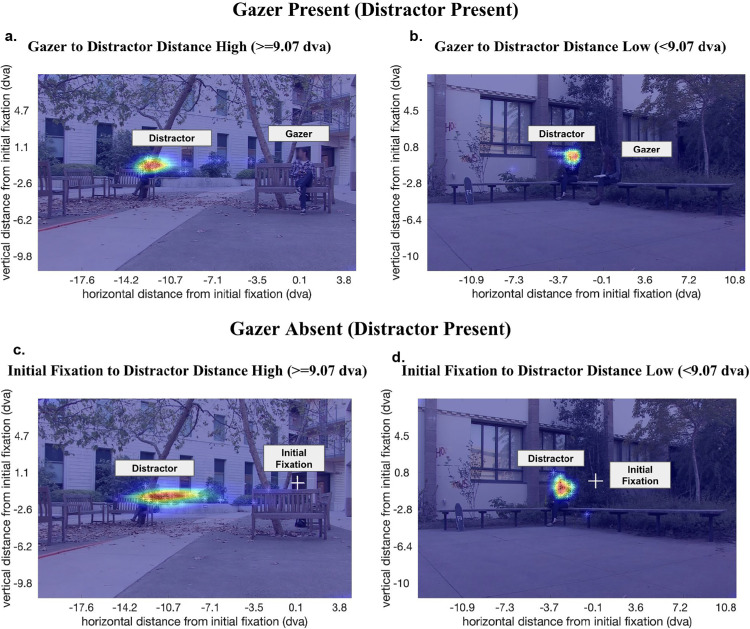
First saccade end point heatmaps. Example trials with the distribution of first saccade end points across all participants showing the presence of the gazer reduces the first saccade error only when the gaze goal (here, distractor) is far (≥9.07 dva). (**a**) Gazer is present and gazer-to-distractor distance is high (≥9.07 dva). (**b**) Gazer is present and gazer-to-distractor distance is low (<9.07 dva). (**c**) Gazer is absent and initial-fixation-to-distractor distance is high (≥9.07 dva). (**d**) Gazer is absent and initial-fixation-to-distractor distance is low (<9.07 dva). Faces in the figure are blurred to protect the privacy of individuals; however, faces were displayed unaltered to the participants of the study.

Even though the presence of a gazer can guide eye movements during visual search, in many real-world circumstances, there might be multiple potential gaze goals that increase the uncertainty about the presence and location of the gazed target. We found that when there are two potential gaze goals in a trial (increased uncertainty), the first saccade end point error is significantly larger than when there is only one (certain) gaze goal location. Our analyses suggest that the increased saccadic error is a consequence of the observer's strategy to fixate the individual closer to the initial fixation (gazer) location first, regardless of whether that person is the goal or the target individual. In other words, when the non-goal is closer to the initial fixation (gazer) location, the observer makes the first saccade to the non-goal thereby leading to an increased distance between the first saccade end point and the goal location than when the goal was present closer to the initial fixation location (see Figure 13, the black diamonds mark the average locations of the non-goal individuals located closer to the initial fixation). These findings can be explained in the light of a cost minimization strategy: when there are multiple potential locations to scan to find the target, one approach may be to program eye movements to each location by starting with the one that is closest to the previous fixation location (Araujo, Kowler, & Pavel, 2001; Kowler, 2011). Importantly, this strategy to fixate the closer individual is observed only in those trials where both the goal and the non-goal were present on the same side of the gazer (95.8% trials). In very few trials (4.2%), the goal and non-goal were present on opposite sides of the gazer and in these trials the goal was located farther from the gazer than the non-goal. Yet, observers first saccades landed on the farther goal on these trials (see Appendix Figures A2 and A3). This highlights the effect of gaze cues in orienting attention toward the gazed direction, and future studies may probe further into the differences between potential goals present on the same side and opposite sides of the gazer.

Previous studies have related features of first saccades to stimulus-level uncertainties in visual tasks such as during visual search. These studies (Beutter, Eckstein, & Stone, 2003, Dickov & Morrison, 2006; Droll et al., 2009; Eckstein et al., 2001, Findlay, 1997; Goliskina et al., 2023; Shimozaki, Schoonveld, & Eckstein, 2012) showed that changes in location uncertainty and stimulus features result in corresponding changes in first saccade latencies and lower accuracy. These results support the intricate link between location uncertainty, perceptual processes, and motor planning. In addition, previous studies have shown evidence for changes in eye movement velocity as a function of expected temporal and spatial uncertainties (Boutachkourt, Drążyk, & Missal, 2024) and have reported slower saccade latencies in the face of spatial unpredictability of target appearance (Dickov & Morrison, 2006).

Our findings add to existing understanding of the influence of uncertainty (multiple likely gaze goals) in saccade planning strategies during visual search in the context of a more ecological task akin to that encountered by the visual system in the real world.

### Observer's first saccades anticipate gazer's head movement

Finally, we found that observers anticipate the gazer's head direction with their first saccade. More specifically, the observer's first saccade predicts the final head direction of the gazer before the gazer finishes their head movement by 100 to 426 ms. This anticipation was present even in the absence of a goal but was more prominent when there were one or more goals present in the periphery. Han and Eckstein (2023b) also made a similar observation, and our study adds to this by showing that the anticipation of gaze is present even when there is more than one potential goal in the visual periphery. Indeed, the anticipation of the gazer's head was more when there was greater uncertainty about true gaze goal location, that is, when more than one likely gaze goals were present in the periphery. A few possible reasons may be cited to explain the greater anticipation with a higher number of potential goals in the visual periphery. First, this was a visual search task, and the stimulus was displayed for a limited time. Further, the target individual was equally likely to be present as both a goal and a non-goal, so the gazer's head direction was not informative about the target location. Taking both these factors into account, observers' higher anticipation (lower saccade latencies) in the presence of two potential goals vs one goal may reflect a time pressure or urgency in the presence of increased uncertainty about the target's location.

Anticipatory saccades have also been found to be involved in motor action planning (Hayhoe & Ballard, 2005; Hayhoe et al., 2003). It is known that eye movements precede some types of motor actions, such as reaching for objects, or picking up objects and placing them elsewhere (Pelz & Canosa, 2001; Schütz et al., 2011; Spering, 2022; Spering et al., 2011). Considering the temporal constraints on eye movement planning, our findings on anticipatory saccades suggest that the brain efficiently uses available information about foveal gazer and peripheral gaze goals to plan goal-driven eye movements.

Importantly, the observers executed first saccades toward the gaze goal (average first saccade angular error = 14.32 ± 0.55°) even when the gazer's head was directed, on average, 60.75 ± 0.73° away from the gaze goal at the time of the first saccade initiation. This finding suggests that the brain integrates information from the periphery with the foveal gazer information to predict the gazer's future direction. Furthermore, the more the available information in the periphery, the greater the anticipation of the gazer's head movement in the observer's first saccade.

Taken together, this study increases our understanding of the fine-scaled temporal dynamics of eye movement programming during visual search with gaze following across naturalistic conditions with varying levels of uncertainty and gaze information. Our study provides evidence that the brain uses dynamic inferential processing to plan eye movement strategies to integrate foveal and peripheral information during gaze following.

## Figures and Tables

**Figure A4.**
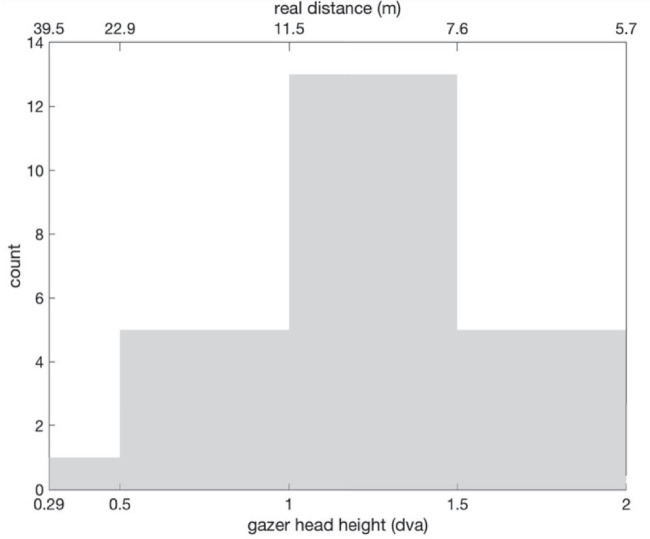
Distribution of gazer head heights and estimated real world distance to the gazer. The bottom axis shows the angle subtended by the gazer's head on the observer's retina and the upper axis shows the estimated real-world distance between the camera and the gazer (actor) that would subsequently result in the corresponding angles subtended, assuming an average head height of 20 cm.

## Appendix

**Table A1.** Gazer influence on the first saccade end point Euclidean error (dva).

First saccade end point euclidean error (dva)						
Condition (A)	Condition (B)	Goal	Mean (A)	Mean (B)	Bootstrap *p* value	Cohen's d
Initial fixation to goal distance high fixation to goal distance high (≥ 9.07 dva)						
Gazer absent	Gazer present	Target	4.34 ± 0.17	3.71 ± 0.19	**<0.001**	0.19
Gazer absent	Gazer present	Distractor	4.47 ± 0.11	4.18 ± 0.12	**0.012**	0.08
Initial fixation to goal distance low Fixation to Goal Distance Low (< 9.07 dva)						
Gazer absent	Gazer present	Target	1.38 ± 0.09	1.26 ± 0.08	0.102	0.1
Gazer absent	Gazer present	Distractor	4.47 ± 0.11	4.18 ± 0.12	0.012	0.08
Gazer present						
Distance to goal						
High	Low	Target	3.71 ± 0.19	1.26 ± 0.08	**0.007**	0.87
High	Low	Distractor	4.18 ± 0.12	1.87 ± 0.07	**<0.001**	0.76
Gazer absent						
Distance to goal						
High	Low	Target	4.34 ± 0.17	1.38 ± 0.09	**<0.001**	1.18
High	Low	Distractor	4.47 ± 0.11	1.93 ± 0.06	**<0.001**	0.93

*Note*: *p* values in bold indicate a significant difference (*p* < 0.05) between compared conditions.

**Table A2.** Gazer influence on the first saccade end point angular error (degrees).

First saccade end point angular error (degrees)						
Condition (A)	Condition (B)	Goal	Mean (A)	Mean (B)	Bootstrap *p*	Cohen's d
Initial fixation to goal distance high (≥ 9.07 dva)						
Gazer absent	Gazer present	Target	6.29 ± 0.31	4.51 ± 0.36	**0.008**	0.3
Gazer absent	Gazer present	Distractor	7.99 ± 0.28	5.79 ± 0.22	**<0.001**	0.28
Initial fixation to goal distance low (<9.07 dva)						
Gazer absent	Gazer present	Target	6.47 ± 0.82	5.09 ± 0.31	0.052	0.16
Gazer absent	Gazer present	Distractor	12.5 ± 0.71	12.41 ± 0.86	0.634	0.01
Gazer present						
Distance to goal						
High	Low	Target	4.51 ± 0.36	5.09 ± 0.31	0.391	0.1
High	Low	Distractor	5.79 ± 0.22	12.41 ± 0.86	**0.006**	0.29
Gazer absent						
Distance to goal						
High	Low	Target	6.29 ± 0.32	6.47 ± 0.82	0.732	0.02
High	Low	Distractor	7.99 ± 0.28	12.5 ± 0.71	**0.021**	0.23

*Note*: *p* values in bold indicate a significant difference (*p* < 0.05) between compared conditions.

**Table A3.** Gazer influence on the first saccade end point amplitude (dva).

First saccade amplitude (dva)						
Condition (A)	Condition (B)	Goal	Mean (A)	Mean (B)	Bootstrap *p*	Cohen's d
Initial fixation to goal distance high (≥ 9.07 dva)						
Gazer absent	Gazer present	Target	10.07 ± 0.13	10.69 ± 0.18	**<0.001**	0.23
Gazer absent	Gazer present	Distractor	8.8 ± 0.09	8.99 ± 0.11	0.09	0.06
Initial fixation to goal distance low (< 9.07 dva)						
Gazer absent	Gazer present	Target	6.01 ± 0.13	6.02 ± 0.15	0.269	0.01
Gazer absent	Gazer present	Distractor	6.18 ± 0.06	5.88 ± 0.07	**<0.001**	0.13
Gazer absent	Gazer present	None	7.56 ± 0.08	9.7 ± 0.12	**<0.001**	0.62
Gazer present						
Distance to goal						
High	Low	Target	10.69 ± 0.18	6.02 ± 0.14	**<0.001**	1.67
High	Low	Distractor	8.99 ± 0.12	5.88 ± 0.07	**<0.001**	1.03
Gazer absent						
Distance to goal						
High	Low	Target	10.07 ± 0.13	6.01 ± 0.13	**<0.001**	1.87
High	Low	Distractor	8.81 ± 0.09	6.18 ± 0.06	**<0.001**	1

*Note*: *p* values in bold indicate a significant difference (*p* < 0.05) between compared conditions.

**Table A4.** Gazer influence on the first saccade onset latency (ms).

First saccade onset latency (ms)						
Condition (A)	Condition (B)	Goal	Mean (A)	Mean (B)	Bootstrap *p*	Cohen's d
Initial fixation to goal distance high (≥ 9.07 dva)						
Gazer absent	Gazer present	Target	196.7 ± 2.73	285.27 ± 6.73	**<0.001**	0.95
Gazer absent	Gazer present	Distractor	186.19 ± 1.48	261.55 ± 4.02	**<0.001**	0.78
Initial fixation to goal distance low (<9.07 dva)						
Gazer absent	Gazer present	Target	193.67 ± 3.15	234.18 ± 6.84	**<0.001**	0.53
Gazer absent	Gazer present	Distractor	196.18 ± 2.23	284.05 ± 4.38	**<0.001**	0.72
Gazer absent	Gazer present	None	272.77 ± 4.67	582.68 ± 11.02	**<0.001**	1.09
Gazer present						
Distance to goal						
High	Low	Target	285.27 ± 6.73	234.18 ± 6.84	0.051	0.45
High	Low	Distractor	261.55 ± 4.02	284.05 ± 4.38	0.11	0.16
Gazer absent						
Distance to goal						
High	Low	Target	196.7 ± 2.73	193.67 ± 3.15	0.48	0.06
High	Low	Distractor	186.19 ± 1.48	196.18 ± 2.23	0.12	0.15

*Note*: *p* values in bold indicate a significant difference (*p* < 0.05) between compared conditions.

**Table A5.** Comparisons between one goal vs two potential goals conditions for the first saccade end point euclidean error (dva).

First saccade end point euclidean error (dva)						
Condition (A)	Condition (B)	Goal	Mean (A)	Mean (B)	Bootstrap *p*	Cohen's d
Gazer present						
One goal	Two potential goals	Target	2.05 ± 0.14	4.19 ± 0.26	**0.014**	0.74
One goal	Two potential goals	Distractor	1.81 ± 0.08	3.85 ± 0.1	**0.005**	0.66
Gazer absent						
One goal	Two potential goals	Target	2.75 ± 0.14	4.03 ± 0.25	**0.021**	0.45
One goal	Two potential goals	Distractor	2.11 ± 0.07	3.89 ± 0.09	**0.002**	0.61
One goal						
Gazer absent	Gazer present	Target	2.75 ± 0.14	2.05 ± 0.14	**<0.001**	0.27
Gazer absent	Gazer present	Distractor	2.11 ± 0.07	1.81 ± 0.08	**0.009**	0.13
Two potential goals						
Gazer absent	Gazer present	Target	4.03 ± 0.25	4.19 ± 0.26	0.286	0.05
Gazer absent	Gazer present	Distractor	3.89 ± 0.09	3.85 ± 0.1	0.321	0.01

*Note*: *p* values in bold indicate a significant difference (*p* < 0.05) between compared conditions.

**Table A6.** Comparisons between goal closer vs goal Farther conditions for the first saccade end point euclidean error (dva).

First saccade end point euclidean error (dva)						
Condition (A)	Condition (B)	Gazer	Mean (A)	Mean (B)	Bootstrap *p*	Cohen's d
Goal: target, non-goal: distractor						
Goal closer	Goal farther	Present	1.06 ± 0.08	5.79 ± 0.29	**<0.001**	1.83
Goal closer	Goal farther	Absent	1.11 ± 0.09	5.54 ± 0.28	**<0.001**	1.78
Goal: distractor, non-goal: target						
Goal closer	Goal farther	Present	1.46 ± 0.12	5.66 ± 0.25	**<0.001**	1.88
Goal closer	Goal farther	Absent	1.39 ± 0.11	5.47 ± 0.23	**<0.001**	1.93
Goal: distractor, non-goal: distractor						
Goal closer	Goal farther	Present	2.19 ± 0.1	6.24 ± 0.18	**<0.001**	1.34
Goal closer	Goal farther	Absent	2.7 ± 0.11	5.68 ± 0.18	**<0.001**	0.98
Goal: distractor, non-goal: distractor						
Gazer absent	Gazer present	Closer	2.7 ± 0.11	2.19 ± 0.1	**<0.001**	0.19
Gazer absent	Gazer present	Farther	5.68 ± 0.18	6.25 ± 0.18	**0.006**	0.16

*Note*: *p* values in bold indicate a significant difference (*p* < 0.05) between compared conditions.

**Table A7.** Gazer-head angular error (degrees).

Gazer-head angular error at the time of first saccade initiation (degrees)					
Condition (A)	Condition (B)	Mean (A)	Mean (B)	Bootstrap *p*	Cohen's d
No gaze-goals	One gaze-goal	48.63 ± 1.44	62.62 ± 1.19	0.12	0.3
No gaze-goals	Two potential gaze-goals	48.63 ± 1.44	69.11 ± 1.13	**0.04**	0.45
One gaze-goal	Two potential gaze-goals	62.62 ± 1.19	69.11 ± 1.13	0.2	0.15

*Note*: *p* values in bold indicate a significant difference (*p* < 0.05) between compared conditions.
